# Sustainable composites based on banana and pomegranate waste incorporated into Polyvinyl Chloride Matrix for methylene blue adsorption

**DOI:** 10.1038/s41598-026-55367-2

**Published:** 2026-06-08

**Authors:** Ahmed Labena, Reda M. Moghazy, Ahmed E. Abdelhamid

**Affiliations:** 1 Egyptian Petroleum Research Institute (EPRI), Nasr City, Cairo, 11727 Egypt; 2 Water Pollution Research Department, National Research Centre, 33 El-Buhouth St, Dokki, Giza, 12622 Egypt; 3 Polymers & Pigments Department, National Research Centre (NRC), 33 El-Buhouth St. Dokki, Giza, 12622 Egypt

**Keywords:** Agricultural waste, Sustainable biosorbents, Composite film, Reusability, Porous materials, Wastewater treatment, Chemistry, Engineering, Environmental sciences, Materials science

## Abstract

The release of synthetic dyes into water bodies presents a major environmental risk, highlighting the need for sustainable treatment methods. Biosorbents derived from agricultural waste demonstrate high effective candidate for dye removal, however their practical application is limited by the challenge of separating of their fine powder from treated water. In this study, eco-friendly, low-cost, and easily recoverable biosorbent materials were developed for the removal of methylene blue (MB) from wastewater. Banana and pomegranate wastes were micro-ground using ball mill, then incorporated into polyvinyl chloride (PVC) (10% agricultural waste relative to the polymer) to form composite films [PVC as a blank, PVC-Banana (PVC-B) and PVC-Pomegranate (PVC-P)] in order to facilitate their harvesting after their usage in the treatment process. Investigation techniques revealed that the synthesized composite films exhibited a highly porous architecture with a porosity ranging from 70% to 73% and a swelling capacity between 472 and 488 wt%. SEM revealed the porous structural of the composite and ART- FTIR revealed successfully incorporated of the agricultural waste within the polymer matrix. Brunauer–Emmett–Teller (BET) analysis revealed remarkably high specific surface areas of 1057 m^2^/g for PVC-B and 531.9 m^2^/g for PVC-P, with pore volumes of 1.90 and 1.08 micrometer, respectively. The composite films were optimized for MB removal by studying the effect of each variable factors via One Factor at a Time (OFAT) followed by factorial design experiments. Results showed that, MB adsorption capacity reached about 75.2 and 66.4 mg/g using 4 g/l PVC-B and PVC-P films, respectively. The adsorption data were best described by the Langmuir isotherm model (R^2^ = 0.997 for PVC-B and 0.998 for PVC-P), with a maximum adsorption capacity (q ^max^) of 79.89 and 70.92 mg/g, respectively. Kinetic studies revealed that the adsorption process followed the pseudo-second-order model (R^2^ ≥ 0.998), suggesting a chemisorption mechanism. The PVC-B and PVC-P displayed good adsorption efficiency and stability in the removal, recovery and reuse of MB for five cycles. Lastly, it can be concluded that the composite films are eco-friendly, cost effective and easily recoverable biosorbents for MB removal.

**Supplementary Information:**

The online version contains supplementary material available at 10.1038/s41598-026-55367-2.

## Introduction

Methylene blue (MB) is a cationic dye that is generated as a waste from different industries such as rubber, plastics, paper, tanning, and pharmaceuticals^1^. These industries discharge their effluents into water streams. This causes severe problems for environment, aquatic & terrestrial living organisms, and human health. The human health-toxic effects of the application of MB are dose-dependent and include serious symptoms such as hemolysis, methemoglobinemia, nausea and vomitus, chest pain, dyspnoea, and hypertension in addition to its carcinogenic and mutagenic effect^2,3^ Therefore, different physicochemical techniques were investigated to help such industries to eliminate the MB from their effluents before discharging^4^. There are many conventional techniques such as membrane-filtration, coagulation, biological oxidation and adsorption that used for elimination of dyes from the industrial discharges^5–8^. However, most of these techniques had a lot of disadvantages, such as their high cost, slow dye removal process, high-energy demand and generation of hazardous wastes after the treatment process^9,10^. Wastewater treatment using agricultural and biological biomasses was another solution that received a lot of interest from researchers due to their availability^11–14^ lower costs and their effectiveness^15,16^. Different biomasses, such as agricultural wastes and microorganisms (bacteria, algae, and fungi) have been previously investigated^17–20^. Banana, orange and pomegranate are types of agricultural wastes that have been used in the dye removal process by many researchers^17,21,22^ Researchers usually collect and grind the biomass to use it as an adsorbent in the wastewater treatment processes. Banana peel biochar and rice husk nanoparticles were used as sustainable adsorbents for dye removal from aqueous solution and showed maximum adsorption capacities of 93 and 94 mg/g for crystal violet (CV), and 94.95 and 75.4 mg/g for MB, respectively^23^. Banana peel waste was converted into porous adsorbents via chemical and thermal activations using sulfuric acid and acetic acid for removal of polar compounds from used palm oil^24^. Pomegranate peel was utilized as a precursor for activated carbon for removal of cadmium (II) ions from aqueous media^25^. Adsorbent hydrogel based on sodium alginate and pomegranate peels were prepared for the removal of Safranin O dye from aqueous solutions and exhibited adsorption capacity of 30.769 mg/g^26^. However, biomasses such as agricultural wastes, usage in powder form is a great challenge for the industrial sector due to their difficulty in harvesting after the treatment process. Therefore, a combined strategy involving the incorporation of agricultural wastes into a polymer substance in the form of film was a good solution for facilitating harvesting^27,28^. Polymers are a type of high molecular weight material that could be organic, inorganic, or organometallic. Different polymer substances, natural or synthetic, such as starch, cellulose, polyethylene, plastics, nylon, and vinyls, could be used^29–32^. Polyvinyl chloride (PVC) is an artificial synthetic polymer that is distinguished by a cheap price and has both chemical and biological resistance^33^. PVC was selected as the matrix material due to its excellent mechanical stability, chemical resistance, and ease of processing, which ensure structural integrity of the composite during adsorption and handling in aqueous media. PVC can be utilized as adsorbent for water treatment after treatment chemically or physically^34,35^ Incorporation of banana and pomegranate wastes within the PVC matrix enables effective immobilization of the biomass, preventing particle leaching while preserving the availability of functional groups such as hydroxyl and carboxyl moieties responsible for methylene blue adsorption. Grinding the agricultural waste, riches with cellulose, hemicellulose, and lignin, along with minor fractions of pectin, proteins, and extractives; these compounds compromising hydroxyl, carboxyl, carbonyl, amine and ester groups, into fine powder will provide functional materials with a high active surface area^36,37^. The incorporation of these functional materials into porous polymer matrix enhances their activity in removal of pollutants. Hence, the aim of the current work is to study the MB removal efficiency using ground agricultural wastes, banana and pomegranate wastes, combined with PVC polymer forming recoverable composite films [PVC as a blank, PVC-Banana (PVC-B) and PVC-Pomegranate (PVC-P)] in order to facilitate their harvesting after their usage in the treatment process. The composite films were further characterized using SEM, ART-FTIR, and BET analyses in addition to porosity and swelling measurements. Afterwards, One factor at a time (OFAT) experiments followed by factorial design experiments were implemented for the optimization process of the composite films in MB removal process. Moreover, adsorption isotherm and kinetics studies were used to demonstrate the mechanism and rate of the adsorption reaction. Then, the optimized agricultural waste films were applied in a process called 3Rs process; removal, recovery and reuse to check their applicability.

## Materials and methods

### Materials

Polyvinyl chloride (PVC, with K value of 60, was purchased from Merck). Dimethyl formamide solvent was delivered from Aldrich. Methylene blue dye (99%) was delivered from Merck. Ethanol, hydrochloric acid (HCl), and sodium hydroxide (NaOH) were in an analytical grade and delivered from Bosch chemical company.

### Sorbent preparation and composite film formation

Banana and pomegranate agricultural wastes were collected from local market (Cairo, Egypt), washed with tap water then with distilled water. The banana and pomegranate peel were cut onto small pieces of approximately 2 x 2 cm^2^ then dried in a drying oven at 90 °C for 24 h. The peels were crushed, finely ground using a ball mill, and subsequently sieved through a 63 µm mesh to obtain particles with sizes below 63 µm. After that, Polyvinyl Chloride (PVC) powder (10 g) was dissolved in (90 ml) Dimethyl formamide at 60 °C for about 4 h till complete dissolution, where the weight percentage of the polymer was 10 wt/v %. The ground banana or pomegranate powders (representing about 10 % of the polymer weight) as active biosorbents were added to the polymer solution and stirred for an hour. The selection of 10 wt% agricultural waste loading in the PVC matrix was based on preliminary screening experiments aimed at balancing adsorption performance, homogeneity of the composite film and mechanical integrity. The obtained mixture solution was casted onto a clean and dried glass plate using a casting knife with a thickness of 250 µm^38^. The casted glass plate was immersed immediately in a non-solvent coagulation bath (water) without any evaporation and left in the water for at least 1 h. Afterwards, the castes glass plate was removed from the bath, washed and stored in water for 24 h then dried in air for further use. Three types of composite films were obtained: polyvinyl chloride (PVC) as a blank film, polyvinyl chloride-banana (PVC-B), and polyvinyl chloride-pomegranate (PVC-P) films.

## Characterization of the composite films

The surface morphology of the tested composite films was examined using scanning electron microscopy (SEM). Prior to the examination, the three types of composite films: PVC, PVC-B, and PVC-P were sputter coated using gold. The corresponding SEM micrographs were obtained using an accelerating voltage of 15 kV (Hitachi SE 900) at 5000× magnification power. The Attenuated Total Reflectance - Fourier Transform Infrared (ART- FTIR) spectrophotometer was used to evaluate the functional groups of the three types of composite films, PVC, PVC-B, and PVC-P. Spectra were assessed within a range of 400–4000 cm^−1^ in a spectrophotometer (Shimadzu). The swelling and porosity of the three types of composite films, PVC, PVC-B, and PVC-P were estimated based on their dry–wet weight procedure^39,40^. The weight of the wetted films was determined after removing the excess water from the film surface. These composite films were dried in a vacuum oven at about 60 °C for 24 h and the dry-weight was determined. The swelling and porosity (P) of the composite films were assessed using the equation 1 and 2, respectively. The specific surface area of the prepared polymer composite was assessed using the Brunauer–Emmett–Teller (BET) technique. Nitrogen adsorption isotherm was measured at 77 K using a Quantachrome TouchWin (version 1.21) surface area analyzer (USA).

1 $$Swelling\left(\mathrm{\%}\right)=\frac{{W}_{wet}-Wdry}{{W}_{dry}}\times 100$$

2 $$P \left( \% \right) = \left[ {\frac{Wwet - Wdr}{{dwAh}}} \right] \times 100$$

Whereas, d_w_ is refers to the pure water density (0.998 g/cm^2^), A is the surface area of the film in the swollen state (cm^2^) and h is the film’s thickness in the swollen state (cm).

## Adsorption process

A stock solution of MB with a concentration of 1000 ppm was prepared by dissolving 1 gm of MB powder in 1 litre distilled water. After that, serial dilutions of 50, 100, 150, 200 and 300 ppm, or according to the needed concentration, were prepared.

Various parameters were optimized through two optimization steps. At first, the effect of each factor was investigated using “One Factor at a Time” to determine the levels that will be included in the second step “full factorial design experiments”.

## Optimization of each factor

Different parameters affecting the adsorption process were evaluated such as contact times (30 - 240 min) pH of MB solution (3–9), film dosage (1–5 g/l), temperature (25–50 °C) and MB dye concentration (50–250 ppm) under shaking condition at 120 rpm. All experiments were conducted in triplicate to ensure data reproducibility. After that, the absorbance readings were taken at 665 nm wavelength for the MB dye by using UV-VIS spectrophotometer (Cary 100 UV–Vis). The following equation was used for the removal efficiency calculation:

3 $$Removal effeciency \left(\mathrm{\%}\right)=\frac{{C}_{o}-{C}_{e}}{{C}_{o}}\times 100$$

Where C_o_ and C_e_ are the initial and final dye concentrations, respectively (mg/l).

## General full factorial design

General full factorial design (2^3^^3^1^) experiments “Many interacted parameters at a time model” were investigated to obtain the optimum conditions that attain high MB removal efficiency^41^. The low and high levels for the optimized factors of the three types of composite films and the design of the matrix of the general full factorial design experiment, including the MB removal efficiency, fits, and residuals were investigated using Minitab18 and the obtained plots Main effects, interaction effects, Pareto chart and response optimizer were interpreted.

## Adsorption isotherms

For the aim of the MB adsorption mechanism determination by using the PVC, PVC-B, and PVC-P composite films as adsorbent materials, the Langmuir and Freundlich, temkin and Dubinin–Radushkevich (D–R) isotherm isotherms were studied as follows:

## Langmuir isotherm

The Langmuir adsorption model was applied to assess the maximum adsorption capability of MB by using the PVC, PVC-B, and PVC-P films. The linear and nonlinear Langmuir isotherm equations were represented according to equation 4 and 5, respectively^42^.

4 $$\frac{Ce}{{Qe}} = \frac{Ce}{{Q\max }} + \frac{1}{{K_{L} Q\max }}$$

5 $${q}_{e}={q}_{m}{K}_{L}\frac{{C}_{e}}{1+{K}_{L}{C}_{e}}$$

Where K_L_ (L/mg) is a constant correlated to the adsorption/desorption capability and Q_max_ (mg/g) is the maximum bio-sorption capacity at complete saturation of the adsorbent film.

## Freundlich isotherm

It’s an empirical equation that used to determine the adsorption intensity of the maximum adsorption capacity of MB using the PVC, PVC-B, and PVC-P films. Linear and nonlinear Freundlich isotherm equations^43^were evaluated using equation 6 and 7, respectively, as follows:

6 $$\mathrm{log}\mathrm{Q}\mathrm{e}=\mathrm{log}\mathrm{K}\mathrm{f}+\frac{1}{\mathrm{n}}\mathrm{log}\mathrm{C}\mathrm{e}$$

7 $${q}_{e}={K}_{f}{{C}_{e}}^{1/n}$$

Where Qe is the adsorption capacity at equilibrium (mg of dye molecules adsorbed/g of films); Ce is the equilibrium concentration of dye in solution (mg/l); K_f_ and n are the Freundlich constants.

## Temkin isotherm

The temkin isotherm^44^ is a model that depends on the pre-assumption that the heat of adsorption would diminish directly with the raise in covering of adsorbent. The linear and nonlinear types of this isotherm were represented by the equations 8 and 9, respectively:

8 $$q_{e} = \left( {{\raise0.7ex\hbox{${RT}$} \!\mathord{\left/ {\vphantom {{RT} {b_{T} }}}\right.\kern-0pt} \!\lower0.7ex\hbox{${b_{T} }$}}} \right)\ln a_{T } + \left( {{\raise0.7ex\hbox{${RT}$} \!\mathord{\left/ {\vphantom {{RT} {b_{T} }}}\right.\kern-0pt} \!\lower0.7ex\hbox{${b_{T} }$}}} \right)\ln C_{e }$$

It can be briefly written as ${q}_{e}=B\mathrm{l}\mathrm{n}{a}_{t}+B\mathrm{l}\mathrm{n}{C}_{e}$ where $B=\frac{RT}{{b}_{T}}$

9 $${q}_{e}=\frac{RT}{{b}_{T}}ln({A}_{T}{C}_{e})$$

Where, ${q}_{e}$ is the amount of adsorbed metal at equilibrium (mg/g); ${b}_{T}$ (mg/L) is the Temkin constant, ${a}_{T}$ (l/g) is a constant at equilibrium binding.

## Dubinin–Radushkevich (D–R) isotherm

The Dubinin–Radushkevich (D–R) isotherm^45^ is a commonly conducted isotherm to estimate the mechanism of the adsorption and assess the porosity properties of the adsorbent in addition to the adsorption apparent energy. The linear and nonlinear equations of D–R isotherm are estimated by equation 10 and 11, as follows:

10 $${\mathrm{l}\mathrm{n}q}_{e}=\mathrm{l}\mathrm{n}{q}_{e}-({K}_{ads}{\varepsilon }^{2})$$

11 $${q}_{e}={q}_{max}exp(-{K}_{d}{\varepsilon }^{2})$$

Where, q_e_ is the hypotheticalv saturation limit (mg/g), Kads is the Dubinin–Radushkevich constant that identified with the mean free energy of adsorption per mole of adsorbate (J/mol) and $\varepsilon$ is the polanyi potential identified with equilibrium and determined by Eq. (12):

12 $$\varepsilon = RT\ln \left( {1 + {\raise0.7ex\hbox{$1$} \!\mathord{\left/ {\vphantom {1 {C_{e} }}}\right.\kern-0pt} \!\lower0.7ex\hbox{${C_{e} }$}}} \right)$$

Where, the universal gas constant (8.314 J/mol K) and the temperature in Kelvin are R and T, respectively. By plotting lnq_e_ versus $\varepsilon$^2^ a straight line formed with a slope equal K_ads_ and intercept equal ln q_e_.

## Kinetics studies

The kinetics of the adsorption process was performed to investigate the rate of the MB adsorption using the PVC, PVC-B, and PVC-P films, which controlled by equilibrium time. The kinetics models involve the pseudo first-order (1^st^) and the pseudo second-order (2^nd^) equations that were applied as follows;

## Pseudo first-order model

The pseudo first-order model linear and nonlinear equations can be calculated as follow^46^:

13 $$\mathrm{l}\mathrm{o}\mathrm{g}({\mathrm{q}}_{e}-{\mathrm{q}}_{t})={\mathrm{logq}}_{e}- \frac{\mathrm{K}1\mathrm{t}}{2.303}$$

14 $${q}_{t}={q}_{e}(1-{e}^{-{K}_{1}t})$$

Whereas qt is adsorption capacity towards MB (mg/g) at a time t, and k1 is the rate constant per minute.

A plot of log (q_e_-q_t_) against time (t) gives a straight line with a slope of (k_1_/2.303) and an intercept of log (q_e_).

## Pseudo second-order model

The data obtained from the effect of time on the adsorption capacity was analysed by the pseudo second-order equations (linear and nonlinear)^47^:

15 $$\frac{t}{{q_{t} }} = \frac{1}{{K_{2} q_{e}^{2} }} + \frac{t}{{q_{e} }}$$

16 $$q_{t} = \frac{{q_{e}^{2} K_{2} t}}{{1 + q_{e} K_{2} t}}$$

Whereas K_2_ is the equilibrium rate constant (g/mg.min). A plot of t/qt against t produces a straight line with a slope of (1/qe) and an intercept of (1/K_2e_^2^).

## Elovich model

The Elovich model is a highly useful model for describing adsorption process on energetically heterogeneous surfaces, where the activation energy increases with the progress of adsorption^48^. The linear and nonlinear forms were assessed as follows:

17 $$q_{t} = \frac{1}{\beta }\ln \left( {\alpha \beta } \right) + \frac{1}{\beta }In(t)$$

18 $${q}_{t}=\frac{1}{\beta }\mathrm{ln}\left(1+\alpha \beta t\right)$$

Where ᾳ (mg/g·min) is the initial adsorption rate and Ꞵ (g/mg) is related to the surface coverage and activation energy.

## Intra-particle diffusion model

To investigate whether the movement of MB into the pores of the micro-ground waste is the bottleneck, this model is used according to following equation.

19 $$q_{t} = \, k_{id} t^{1/2} + \, C$$

Where k_id_ (mg/g·min^0.5^) is the intraparticle diffusion rate constant and C (mg/g) represents the boundary layer thickness. If C = 0, intraparticle diffusion is the sole rate-limiting step; if C > 0, multiple mechanisms are at play.

## Thermodynamic study

Thermodynamic analysis of the MB adsorption process was conducted to assess the nature of the adsorption mechanisms and to evaluate the observed temperature-dependent behaviour. The Gibbs free energy change of the adsorption reaction is related to the distribution coefficient (Kd) values. The natural logarithm of Kd was plotted against the reciprocal of absolute temperature (1/T) according to the equation:

20 $$\ln \left( {Kd} \right) \, = \, - \Delta H^\circ /\left( {RT} \right) \, + \, \Delta S^\circ /R$$

where ΔH° is the standard enthalpy change, ΔS° is the standard entropy change, R is the universal gas constant (8.314 J/(mol•K)), and T is the absolute temperature in Kelvin. Linear regression analysis of the Van’t Hoff plot yielded ΔH° from the slope and ΔS° from the y-intercept. The standard Gibbs free energy change at 298.15 K (25°C) was subsequently calculated using the fundamental relationship: ΔG° = ΔH° - TΔS°. Additionally, a ΔG° versus temperature plot was constructed to derive independent estimates of both ΔH° and ΔS° values, providing evaluation of the calculated parameters.

## Desorption study

After implementation of the optimization process, the optimum factors were reinvestigated to confirm the result of the response optimizer and the performance of the reuse-experiment. Aqueous ethanol concentrations of 50 % were applied to investigate the reusability of the selected films. The composite film after adsorption of methylene blue was soaked in the aqueous ethanol solution under stirring for about 30 min. then decanted and repeated the washing twice then the film was dried and enter in another adsorption cycle. The MB removal efficiency of the composite after washing was determined with relative to the initial efficiency.

## Results and discussion

### Characterization of the composite films

The prepared composite films were characterized using different methods such as SEM, ART-FTIR, swelling and porosity measurements, and the results were presented as follows:

### SEM analysis

**Fig. 1:**
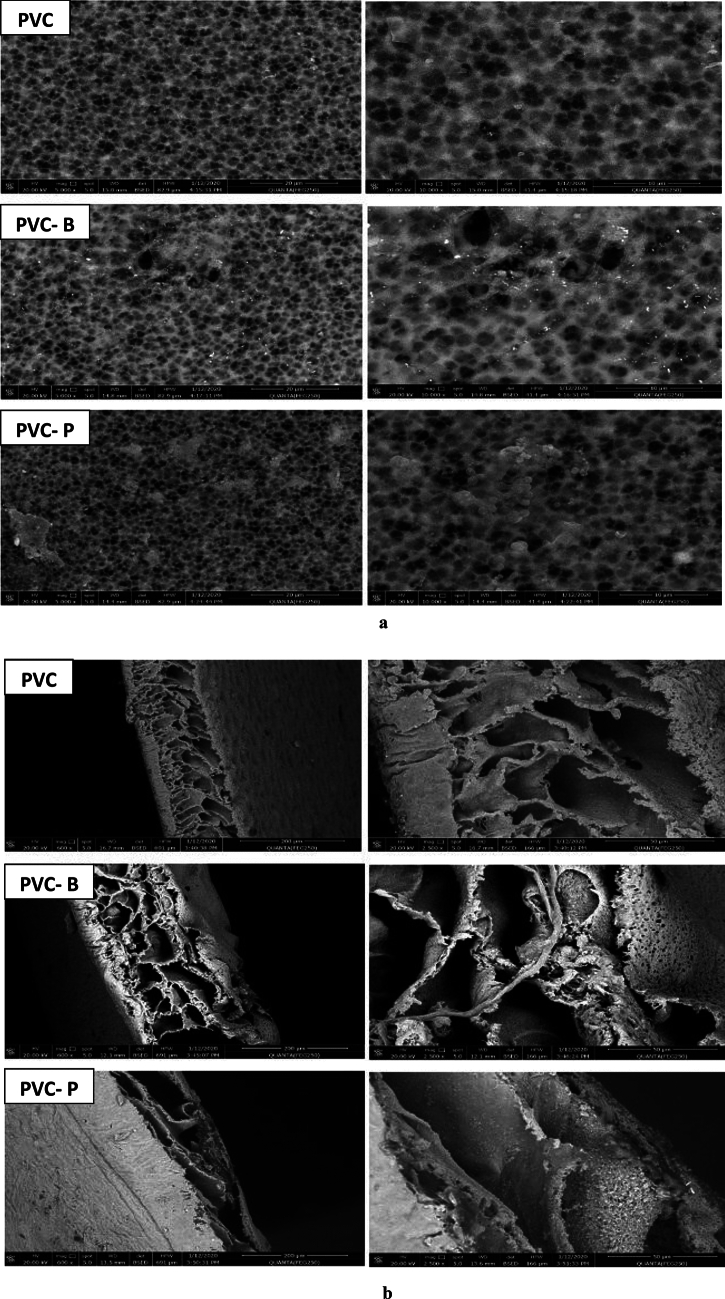
SEM analysis of the PVC, PVC- B and PVC-P films surface at two different magnifications (5000x left and 1000x right).

The surface and cross-sectional morphologies of the prepared films were investigated via SEM, as shown in Fig. 1 (a & b). The blank PVC film exhibits a typical asymmetric structure characterized by a thin dense top layer and a highly porous, sponge-like sub-layer with macro-voids, which is characteristic of the phase inversion technique. Upon the incorporation of agricultural wastes, the particles of banana and pomegranate appear successfully embedded within the polymer matrix and inside the pores. At higher magnifications, these particles appear as spherical balls distributed on the surface.

The observed variations in pore density and interconnectivity between PVC-B and PVC-P can be attributed to the inherent chemical composition and hydrophilicity of each additive. Banana peels, being richer in hydrophilic pectin and polysaccharides, increase the affinity of the casting solution toward the water bath. This accelerates the solvent/non-solvent exchange rate during the demixing process, leading to the more developed and interconnected porous network observed in PVC-B compared to PVC-P. This intensified porosity, confirmed by the higher BET surface area of PVC-B, directly correlates with its superior adsorption performance, as it provides easier pathways for Methylene Blue molecules to reach the active binding sites^49^.. The macro-void formed due to the thermodynamically instantaneous de-mixing of the PVC casted solution in the coagulation bath. The PVC-P film showed a highly macro-void structure with the presence of some relatively unground particles. For the PVC-B film, the cross-section also showed a highly macro-void with micro globules especially at a high magnification power.

### ART- FTIR analysis

**Fig. 2:**
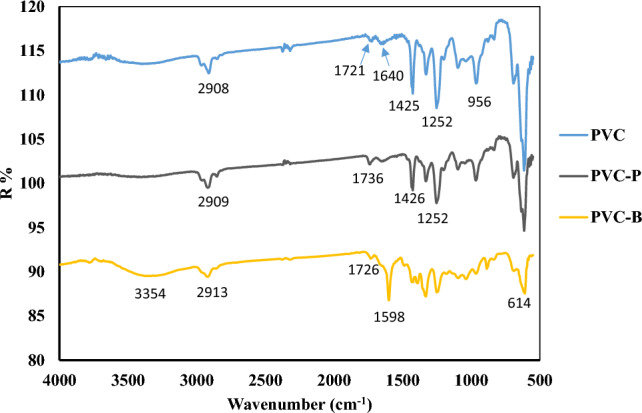
ATR-FTIR of the PVC, PVC- B and PVC-P films.

The ATR-FTIR spectra of the prepared films are illustrated in Fig. 2. For the blank PVC film, characteristic absorption peaks were observed at 1425 and 1327 cm^−1^, corresponding to C-H bending and C-H rocking, respectively. The peaks at 960 and 614 cm^−1^ were attributed to C-Cl stretching and bending vibrations, respectively, while the peak at 2908 cm^−1^ related to C-H stretching^39,50^. The presence of peaks at 1250 and 1730 cm^−1^ (C-O and C=O stretching) in the blank film may indicate the minor oxidation or additives of the polymer. It also showed characteristics peaks at 1250 and 1730 cm^−1^ which attributed to C-O and C=O stretching bands, respectively, that may be indicated the oxidation and additives in PVC polymer. Upon the incorporation of banana and pomegranate wastes, significant changes were observed, confirming the integration of lignocellulosic components. The broad peak around 3354 cm^−1^ is attributed to the O-H stretching vibrations of cellulose and hemicellulose. The absorption peak at 1735 cm^−1^ significantly increased in intensity, which is specifically assigned to the C=O stretching of esterified carboxyl groups from pectin, hemicellulose, and lignin. Furthermore, the strong peaks in the 1600–1660 cm^−1^ region correspond to the aromatic C=C skeletal vibrations and C=N groups, indicating the presence of lignin and phenolic compounds from the agro-wastes^51^. Based on the FTIR analysis, both additives contribute an abundance of hydroxyl (-OH) and carboxyl (-COOH) groups. These groups serve as active binding sites that enhance the adsorption capacity through electrostatic attraction and hydrogen bonding with the cationic Methylene Blue (MB) molecules. The observed difference in performance—where PVC-B outperforms PVC-P—is rooted in the distinct biochemical composition of the two additives. Banana peels are exceptionally rich in pectic polysaccharides and cellulose, which possess a high density of accessible carboxyl and hydroxyl groups. These components not only provide more binding sites but also increase the film’s hydrophilicity, promoting better swelling and easier access for the dye molecules. In contrast, pomegranate waste is characterized by a higher content of lignin and condensed tannins. The complex, cross-linked aromatic structure of lignin can ‘mask’ some of the functional groups, making them less accessible for sequestration. Consequently, the pectin-rich nature of banana waste provides a more favorable chemical environment for MB binding than the more lignified framework of pomegranate waste^52–54^

### Swelling and porosity

The swelling and porosity of the prepared composite films, before and after incorporation of the agricultural wastes were demonstrated in Table 1**.** The swelling of the composite films is an indicator for the hydrophilic nature of the films. This makes the water with the contaminant easily absorbed and contacted with the functional groups within the films’ matrix, which results in more chance for pollutant removal. The swelling capacity of the composite films increased from 460% for the blank PVC to 472% and 488% for the PVC-P and PVC-B films, respectively. This increment is primarily attributed to the hydrophilic nature of the incorporated agro-wastes, which are rich in polysaccharides bearing reactive functional groups such as hydroxyl (-OH) units^55^. The higher swelling of PVC-B (488%) compared to PVC-P (472%) lies in the contrasting chemical compositions of the additives. Banana peels possess a higher content of pectic polysaccharides and cellulose, which are inherently more hydrophilic and promote greater water entrapment. In contrast, pomegranate peels are richer in lignin and tannins; these polyphenolic compounds have a more complex, cross-linked, and relatively hydrophobic aromatic structure that can restrict the degree of matrix expansion.

**Table 1:** Swelling and porosity calculations.

**Parameters/Films**	**PVC**	**PVC- P**	**PVC-B**
Swelling (%)	460	418	488
Porosity (%)	70	72	73

Furthermore, the porosity of all prepared films exhibited high values, ranging from 70% to 73%. This high porosity facilitates the rapid diffusion of water-loaded contaminants into the polymer matrix, providing a large internal surface area for adsorption. Notably, the enhancement in swelling and porosity was relatively moderate. This is because the blank PVC itself possesses a highly developed, sponge-like porous structure that already traps a significant volume of water. The addition of waste particles—while providing more active functional groups—also results in the partial occupation of these existing macro-voids, leading to the observed balanced enhancement in the film’s physical properties.

### Brunauer–Emmett–Teller (BET) analysis

**Fig. 3:**
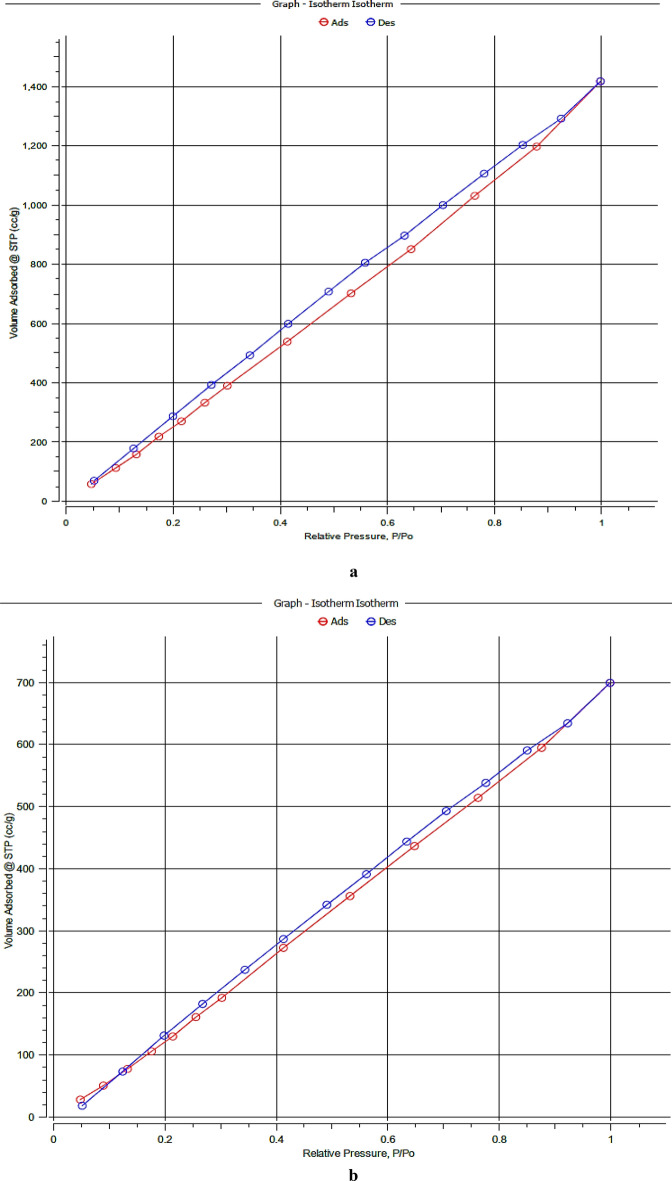
BET surface area for (**a**) PVC-B and (**b**) PVC-P composite films.

The BET analysis was performed to evaluate the specific surface area of the prepared composite films. The nitrogen adsorption–desorption isotherms for the PVC-B and PVC-P films were displayed in Fig. 3a and b, respectively. The figure demonstrated the significant nitrogen adsorption that indicating their ability for enhanced adsorption efficiency^56^. The analysis reported that the surface area for the PVC-B film was 1057 m^2^/g with a total pore volume of 1.9054 cc/g and an average pore radius of 2.1964 nm for pores smaller than 421.97 nm at a relative pressure of 0.9977. While the surface area for the PVC-P film was 531.9 m^2^/g and the total pore volume 1.0838 cc/g with an average pore radius of 1.925 nm for pores smaller than 857.5 nm at a relative pressure of 0.9988. The high surface area of the two composite films indicates the suitability of these films for improved adsorption process. The high porosity of these polymer composites makes them promising candidates for application in remediation of pollutants such as dyes from water. The higher surface area of the PVC-B than PVC-P film reflects the high potential of the PVC-B film in the adsorption process with a high efficiency.

The inclusion of banana and pomegranate particles resulted in a significant expansion of the Specific Surface Area (SSA). According to the IUPAC classification, the nitrogen adsorption-desorption isotherms for the composite films followed a Type IV pattern with an H3 hysteresis loop, confirming the mesoporous nature of the material. The rationale for this enhancement lies in the interaction between the biomass and the PVC matrix during film formation; the hydrophilic particles accelerate the de-mixing process, creating larger and more numerous macro and meso-voids. Previous reports on banana and pomegranate peels have shown varied BET results, often reaching high values when integrated into polymer matrices or after chemical activation^57,58^ The average pore diameter calculated for PVC-B and PVC-P was found to be in the range of 10–25 nm, confirming a mesoporous characteristic. This predominance of mesoporosity (pore diameter range 2–50 nm) is particularly advantageous for the sequestration of Methylene Blue. Given that the molecular dimensions of the MB cation are approximately 1.43 nm X 0.61 nm X 0.4 nm, these wide mesoporous channels provide low-resistance pathways for the dye molecules to diffuse deeply into the internal active sites of the composite matrix. Consequently, this architecture effectively overcomes the steric hindrance and mass transfer limitations often encountered in purely microporous materials, explaining the superior adsorption kinetics observed in this study.

**Table 2:** Adsorption isotherm parameters.

Isotherm model	Mode/representation	Parameter	Blank PVC	PVC-B (Banana)	PVC-P (Pomegranate)
Langmuir	Linear Mode	Q^max^ (mg/g)	7.7326	79.8909	70.9225
		K_L_(L/mg)	227659	0.3171	0.2033
		R^2^	0.6521	0.9971	0.9984
	Non-linear Mode	Q^max^(mg/g)	*5.1200*	*81.2400*	*72.1500*
		K_L_(L/mg)	*0.0125*	*0.2850*	*0.1910*
		R^2^	0.6125	0.9113	0.8954
Freundlich	Linear Mode	K_F_(mg/g)(L/mg)^1/n^	97.1839	41.0996	27.9168
		1/n	−2.2830	7.0000	5.0324
		R^2^	0.2690	0.8980	0.8384
	Non-linear Mode	*K*_F_	*12.4500*	*43.1200*	*29.4500*
		1/n	*−1.1500*	*6.5400*	*4.8800*
		R^2^	0.2104	0.7845	0.7412
Temkin	Linear Mode	A_T_ (L/mg)	0.0002	86.6479	6.8880
		(J/mol) b_T_	−621.71	285.8867	234.1168
		R^2^	0.1332	0.9061	0.8514
	Non-linear Mode	*A*_T_(L/mg)	*0.0001*	*82.1500*	*5.9200*
		*b*_T_ (J/mol)	*−412.50*	*261.4000*	*211.8000*
	Linear Mode	R^2^	0.1015	0.6508	0.6122
Dubinin–Radushkevich (D-R)		q_s_(mg/g)	13.5052	75.5676	68.0854
		E (J/mol)	829084	668.5086	246.2840
		R^2^	0.5512	0.9778	0.9980
	Non-linear Mode	q_s_(mg/g)	*9.4500*	*76.1200*	*69.1100*
		E(J/mol)	*745120*	*642.1500*	*231.4500*
		R^2^	0.5114	0.9615	0.9712

## Adsorption process optimization

### Effect of each factor

The effect of each factor on the MB removal efficiency was studied, and the results will be explained in the following sections. Optimum levels were utilized to assess the low and high levels of each parameter that was included further in the full factorial investigation. The concentration of MB before and after adsorption process was measured using UV-Vis spectroscopy at wavelength 660 nm using the calibration curve displayed in Fig (S1).

**Table 3:** Kinetics parameters.

**Film type**	**Model**	**R** ^2^	**qe​ (mg/g)**	**k1​ or k2​**	**α**	**β**	**kid​**	**C**
Blank PVC	Elovich (L)	0.959	-	-	0.213	0.181	-	-
	IPD (NL/L)	0.927	-	-	-	-	1.118	−4.494
	PFO (NL)	0.915	18.21	0.0047	-	-	-	-
PVC-B	Elovich (L)	0.945	-	-	0.565	0.123	-	-
	PFO (NL)	0.942	25.03	0.0099	-	-	-	-
	PSO (NL)	0.942	35.32	0.0002	-	-	-	-
	IPD (NL/L)	0.930	-	-	-	-	1.661	−1.983
PVC-P	PSO (L)	0.992	15.34	0.0021	-	-	-	-
	Elovich (NL)	0.928	-	-	1.660	0.363	-	-
	IPD (NL/L)	0.920	-	-	-	-	0.553	5.445

### Effect of contact time

**Fig. 4:**
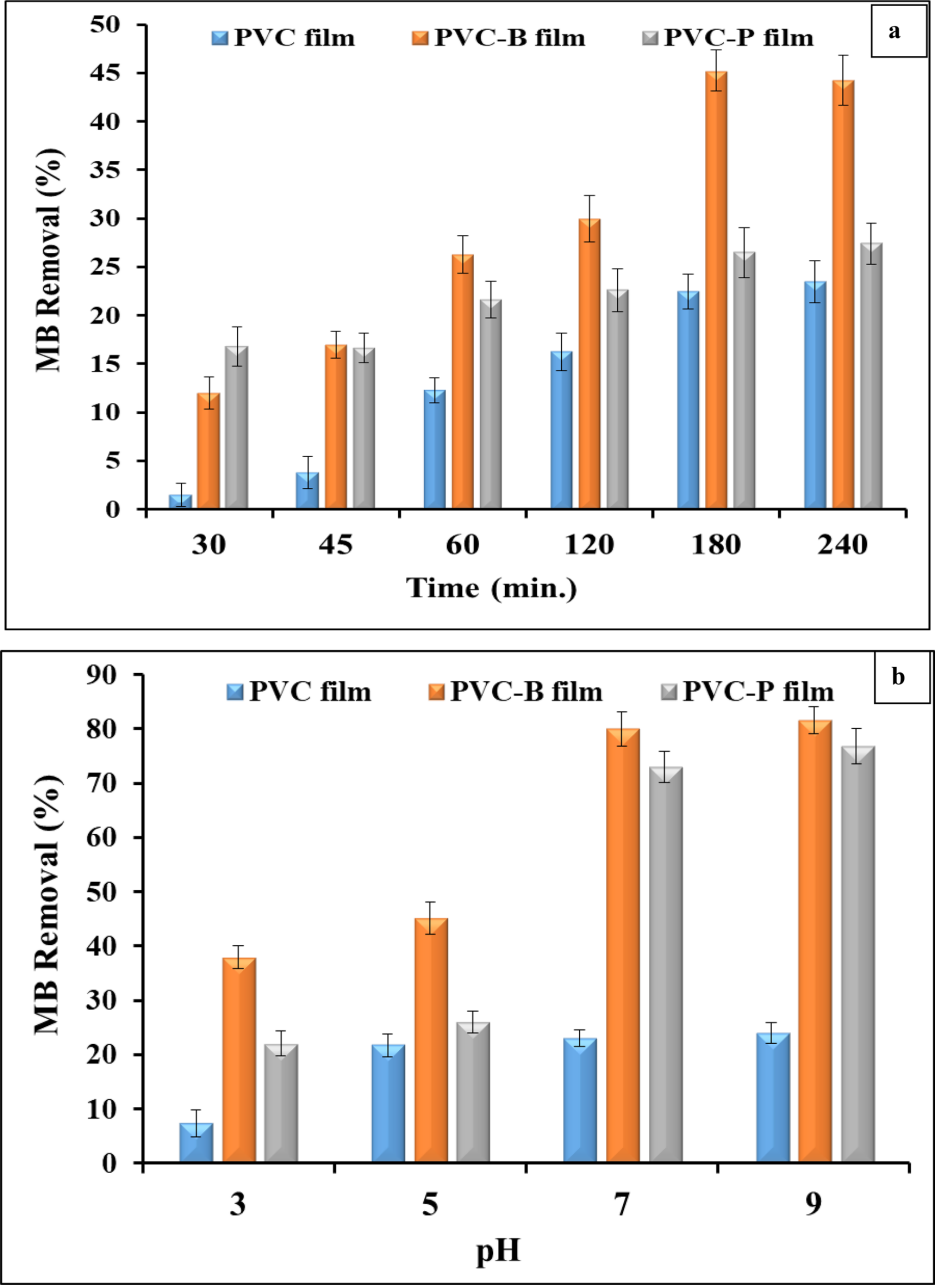
Effect of (**a**) contact time and (**b**) pH of MB solution on the removal efficiency.

"The effect of contact time on MB adsorption by PVC, PVC-B, and PVC-P films is illustrated in Fig. 4b. Results demonstrated that the PVC-B film recorded the highest removal efficiency, reaching nearly 45.2% after 180 min, followed by PVC-P and the blank PVC. The adsorption process for all films exhibited a rapid initial uptake, which can be attributed to the enormous number of vacant active sites (such as hydroxyl and carboxyl groups) that are highly accessible in the early stages, thereby increasing the adsorption rate due to the high concentration gradient.

The significant superiority of PVC-B over PVC-P in terms of both rate and capacity is fundamentally linked to the additive’s nature. As discussed in the SEM and BET sections, the higher hydrophilicity of banana peels (richer in pectin and cellulose) compared to the more lignified pomegranate peels promotes faster swelling and better wetting of the film. This reduces mass transfer resistance, allowing MB molecules to penetrate the internal matrix more effectively^59^.

Furthermore, the highly developed mesoporous structure of PVC-B provides a larger effective surface area for the dye molecules (1.43 nm X 0.61 nm) to diffuse into. Over time, as these active sites become occupied and the surface concentration of dye particles increases, the electrostatic repulsion between the adsorbed dye and the bulk molecules, along with the saturation of internal pores, leads to the observed plateau at 180 min. This equilibrium state signifies that the adsorption process shifts from rapid surface binding to a slower, pore-diffusion controlled mechanism, eventually becoming less favorable as the system reaches saturation^60^.

**Table 4:** Thermodynamic parameters for MB adsorption on PVC-based films.

**Biosorbent**	**ΔHº** ** (kJ/mol)**	**ΔSº (J/mol·K)**	**ΔGº (kJ/mol) at 298.15 K**	**ΔGº** ** (kJ/mol) at 308.15 K**	**ΔGº (kJ/mol) at 318.15 K**	**R (Van 't Hoff)** ^2^
PVC	−9.63	−11.4	−6.23	−6.12	−6.01	0.9982
PVC-B	−12.71	−19	−7.05	−6.86	−6.67	0.9974
PVC-P	−17.2	−30.79	−8.02	−7.72	−7.41	0.9958

### Effect of pH

The pH plot was presented in **Fig (4-c)** where the highest MB removal efficiency was achieved at basic conditions at pH 7 and 9, with percentages of nearly 79.9 and 81 % for the PVC-B, respectively. The PVC-P film displayed a removal efficiency of 73 and 76.8% at pH 7 and 9, respectively. Whereas, there is no much difference was noticed in the change value for the blank PVC film, where the removal efficiency ranging from 21 to 24 % at the pH range 5 - 9. Moreover, the PVC-B film recorded the highest MB removal efficiency at pH 7 and 9 and this results could be explained by, the increase in pH of solution, the increasing the negative charges of the functional groups of the cell wall of the agricultural wastes such as OH^-^ and COO^-^ that help in the removal of cationic MB dye^61^. This result could be confirmed by the study that was performed on the Congo red removal by the PVA/SA/ZSM-5 zeolite membrane, whereby increasing pH of the anionic Congo red, dye removal efficiency decreases due to the increase in negative charges^62^. Nevertheless, in the acidic conditions, the protonated functional groups that carry positive charges did not interact with the same charges leading to a decrease in the adsorption process. The results indicated that there are no large difference in the removal efficiency between pH 7 and 9, therefore the following experiments was performed at pH 7.

### Effect of film dosage

**Fig. 5:**
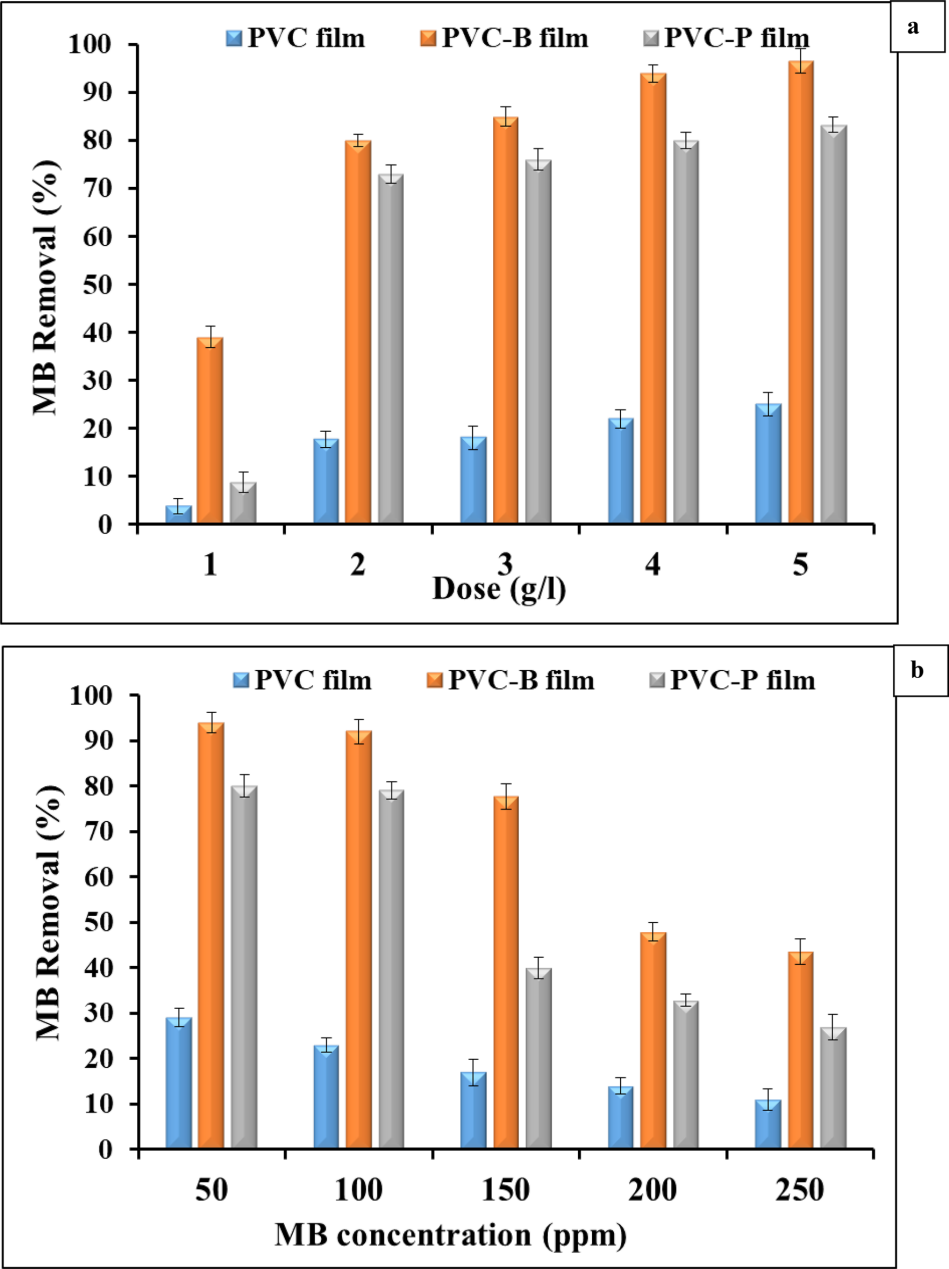

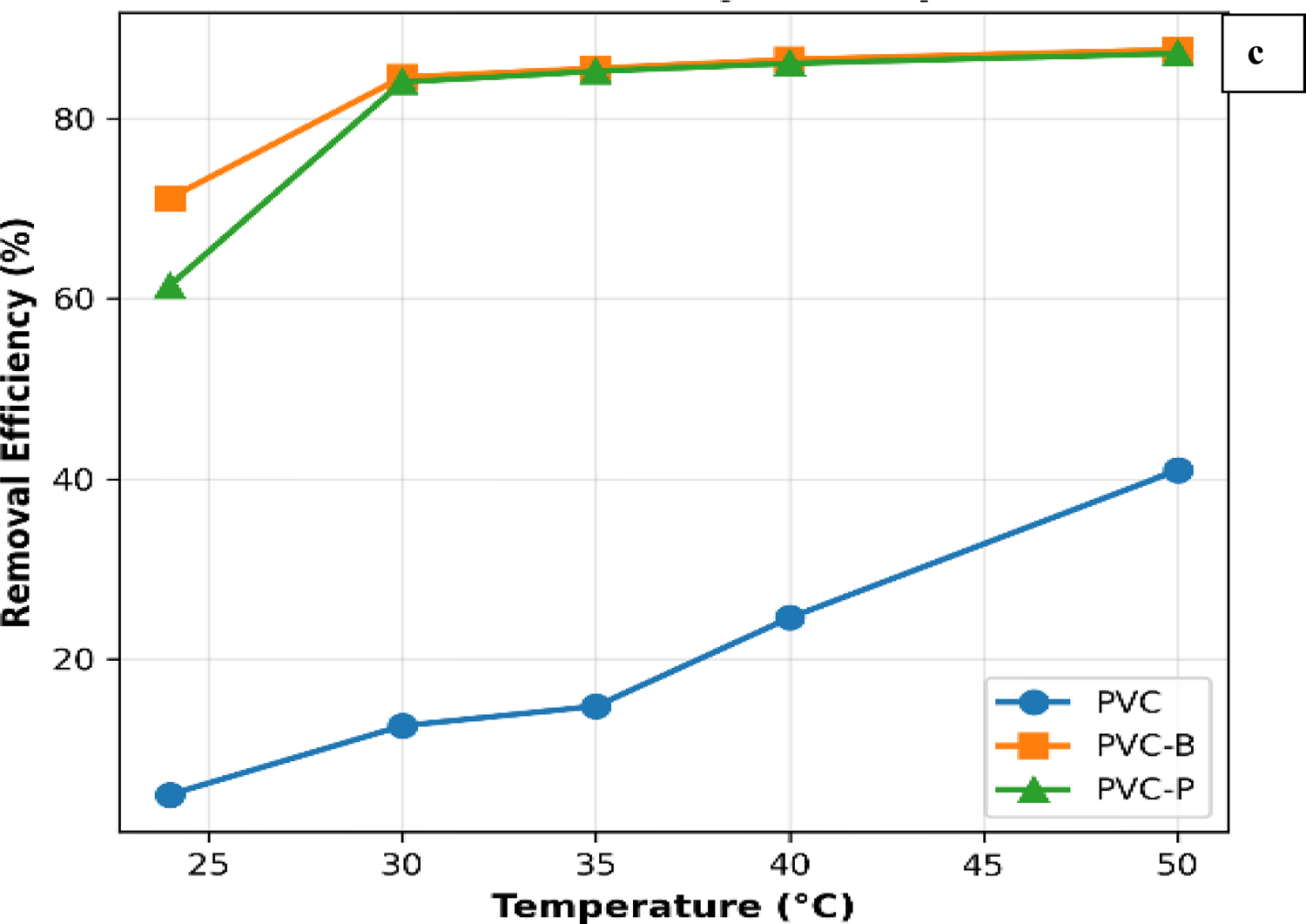
Effect of (**a**) dose of film, (**b**) MB concentration and (**c**) Temperature on the MB on the removal efficiency.

It was noticed that the MB removal efficacy improved by increasing the film dosage from 1 to 4 g/l, after that, it recorded nearly constant values by using 5 g/l (see Fig 5a**)**. Additionally, the PVC-B film recorded the highest MB removal efficiency at 4 g/l after 180 min contact time, which was nearly 94 % with adsorption capacity (qe) of 79.89 mg/g. PVC-P recorded removal efficiency of 80% with adsorption capacity (qe) of 70.92 mg/g. Increasing the adsorbent dosage increases the surface including the active sites that achieve a higher removal percentage of the pollutant molecules, such as MB^39,63^. The increase in the adsorption efficiency by increasing the adsorption dosage is a reasonable result due to the availability of more reactive functional groups for the adsorption process, however, the increase in adsorbent dosage led to a decrease in the adsorption capacity, as capacity related to amount of adsorbed dye per amount of adsorbent, while efficiency related to the decline of the MB in solution regardless of the amount or dosage of adsorbent. This was reported previously in some research, such as the effectiveness of adsorption for the three dyes: for Acid Blue 25, Acid Orange and Acid Black 1, increased to achieve 93.7%, 95.6% and 87.1% by increasing the dosage up to 3g/l^64^.

### Effect of methylene blue initial concentration

The effect of the initial MB concentration on the adsorption performance was evaluated and displayed in Fig. 5b. Results indicated that the removal efficiency (RE%) for PVC-B, PVC-P, and blank PVC reached approximately 94%, 80%, and 29%, respectively, at a 50 ppm dye concentration after 180 min of contact time. Notably, the PVC-B film demonstrated the highest performance, with its removal efficiency slightly decreasing from 94% at 50 ppm to 92% at 100 ppm with adsorption capacity increment to 79.89 mg/g. It is clarified that while the removal efficiency (RE%) decreases at higher concentrations (above 100 ppm), the adsorption capacity (q_e_) representing the amount of dye adsorbed per unit mass—actually increases. This increase in q_e_ is attributed to the higher concentration gradient, which provides a stronger driving force to overcome the mass transfer resistance. The observed downward trend in removal efficiency as concentration increases is due to the progressive saturation of the adsorbent’s active sites; as the number of MB molecules increases, the available sites on the composite films become occupied until no further molecules can be sequestered. This behaviour is consistent with previous studies on MB removal using biomass-based adsorbents (e.g., Ref 60), where the ratio of active sites to dye molecules decreases at elevated initial concentration^65^. This result agreed well with the result in the previous studies of MB removal using activated carbon of banana peel^66^.

This superior performance of PVC-B, reaching a removal efficiency of 94%, is not merely a numerical result but is deeply rooted in the synergistic combination of its chemical and structural properties, Chemically, banana peels are exceptionally rich in pectic substances and cellulose, which provide a higher density of hydroxyl and carboxyl functional groups compared to the more lignified pomegranate peels. These groups act as primary anchoring sites for the cationic Methylene Blue molecules via electrostatic interactions.Structurally, the PVC-B film exhibited a significantly higher Specific Surface Area (1057 m^2^/g) and a more interconnected mesoporous network, as confirmed by BET and SEM analyses. This architecture, combined with the superior hydrophilicity and swelling capacity of the banana-waste matrix, minimizes mass transfer resistance and ensures that a larger fraction of the dye molecules can reach the internal active sites within the 180-minute contact period, leading to the observed 94% removal efficiency.

### Effect of temperature

The effect of temperature on the removal efficiency and adsorption capacity of the three biosorbent films is presented Fig. 5**-c**. As temperature increased from 24 °C to 50 °C, all three composite films enhanced the adsorption efficiency, with distinctive patterns that reflect the endothermic nature of the overall adsorption process. For the pristine PVC biosorbent, the removal efficiency increased from 4.92% at 24 °C to 40.98% at 50 °C, representing an absolute increase of 36.07%. This substantial temperature-dependent enhancement suggests that the adsorption process requires thermal energy to overcome activation barriers. The equilibrium concentration decreased from 87 mg/l to 54 mg/l, and the adsorbed quantity increased from 4.5 mg/g to 37.5 mg/g over this temperature range. The distribution coefficient (Kd) exhibited a marked increase from 0.0517 to 0.6944, indicating progressive improvement in the affinity of the adsorbent for the MB molecules as temperature increases.

The modified PVC-B film displayed significantly superior performance compared to pristine PVC at all examined temperatures. At 24 °C, the PVC-B film achieved a removal efficiency of 71.15%, which improved to 87.65% at 50 °C, representing a more modest increase of 16.50% in absolute terms. This suggests that the banana waste incorporation provides inherent favourable binding sites that function effectively even at lower temperatures. The Ce values decreased rapidly from 26.4 mg/l at 24 °C to 11.3 mg/l at 50 °C, indicating increasingly efficient removal of the MB from the solution. The adsorption capacity (qe) ranged from 65.1 mg/g to 80.2 mg/g, demonstrating a substantial improvement conferred by the banana waste modification. The distribution coefficient showed a progressive increase from 2.4659 to 7.0973, reflecting the enhanced and temperature-responsive binding characteristics of this material.

The PVC-P film, incorporating pomegranate waste, exhibited an intermediate performance between the PVC and the PVC-B films. At 24 °C, the removal efficiency was 61.42%, increasing to 87.21% at 50 °C, with an absolute increase of 25.79%. The equilibrium concentrations decreased from 35.3 mg/l to 11.7 mg/l, and the adsorption capacity increased from 56.2 mg/g to 79.8 mg/g. The Kd values ranged from 1.5921 to 6.8205, demonstrating temperature-responsive improvement in adsorbent efficiency. These results indicated that, the pomegranate waste component contributes favourable functional groups and structural properties that enhance the MB binding, though to a lesser extent than the banana waste modification. The temperature-dependent behaviour observed across all three composite films is consistent with an endothermic adsorption process where higher temperatures facilitate increase molecular motion and better diffusion of the MB molecules into the biosorbent matrix. The convergence of removal efficiencies at higher temperatures (approximately 87% for both modified materials) suggested that the limiting factor at elevated temperatures becomes the saturation of available binding sites rather than kinetic limitations.

### Factorial design

The efficiency of methylene blue (MB) removal using PVC-based films (PVC, PVC-B, and PVC-P) was assessed via a full factorial design encompassing five key factors: MB concentration (50–300 mg/L), adsorbent dosage (1–4 g), contact time (30–180 min), temperature (30–50°C), and pH (3–9). This approach employed ordinary least squares (OLS) regression models, supplemented by Pareto charts and response surface plots, to assess the multifaceted interactions governing adsorption performance.

### Statistical reliability and model performance

The OLS models derived from the 24-run ${2}^{3}\times {3}^{1}$ factorial dataset demonstrated a robust performance across PVC, PVC-B, and PVC-P films, with ${R}^{2}=95.49\mathrm{\%}$ indicating the factors explained of 95.5% of the MB removal variance. Adjusted ${R}^{2}=89.64\mathrm{\%}$ confirmed fit quality despite multiple terms, while the predicted ${R}^{2}=74.05\mathrm{\%}$ (LOOCV-equivalent) and the root mean square error $S=9.74\mathrm{\%}$ validated generalization to unseen data. Fitted values (FITS) mirrored observations closely in the most runs (e.g., Run 20: 95.64% observed vs. 93.67% fitted; Run 12: 94.90% vs. 95.93%), though outliers like Run 22 (−15.95 RESI) underscored dosage limitations at the high MB concentration (300 ppm)/low dosage (1 g/l). These metrics, bolstered by the expanded temperature/pH ranges in the full design, enhance a predictive fidelity for the adsorption processes.

### Main effects analysis

Main effects plots revealed consistent trends (see **Fig S2**), underscoring the optimization pathways. Adsorbent dosage emerged as the paramount factor per the Pareto analysis, with a removal efficiency rising sharply from 1 to 4 g due to greater availability of active sites and a pattern well-documented in biomass-based adsorbents. Temperature and pH displayed positive correlations; elevated temperatures (50°C) likely enhance endothermic adsorption or molecular diffusion, while at alkaline pH (9) promotes an electrostatic attraction for the cationic MB via the surface deprotonation. Conversely, higher MB concentrations (up to 300 mg/l) reduced percentage removal, reflecting a site saturation, and extended contact times (up to 180 min) drove equilibrium attainment.

### Interaction effects

Two-way interaction plots evidenced non-additive effects (Fig S3), with non-parallel lines indicating synergies. Dosage-pH interactions amplified benefits at a high pH, optimizing surface charge; dosage-temperature pairings further boosted efficiency, suggestive of thermodynamically favoured binding. The contact time-temperature synergies implied accelerated kinetics at 50 °C, while the MB concentration’s adverse impact was attenuated by elevated dosage or pH tuning.

### Pareto analysis of factor significance

Pareto charts ranked effects by standardized coefficients (Fig S4), confirming dosage as dominant across films, followed by temperature, contact time, pH, and key interactions (e.g., dosage-temperature, dosage-pH). This hierarchy mirrors complex adsorption dynamics in polymeric systems.

### Adsorption isotherm

The equilibrium data for MB adsorption onto PVC-B and PVC-P composite films were assessed using Langmuir, Freundlich, Temkin, and Dubinin–Radushkevich (D-R) models to elucidate the adsorption mechanism. As displayed in Fig (S5 & S6) and summarized in Table 2, both composite films exhibited excellent correlation with the Langmuir and D-R models, with regression coefficients (R^2^) exceeding 0.99. Specifically, the Langmuir model yielded R^2^ values of 0.9971 and 0.9984 for PVC-B and PVC-P, respectively, while the D-R model recorded 0.9778 and 0.9980. The superior fit of the Langmuir model suggests that the adsorption occurs via monolayer formation onto a surface with a finite number of identical and energetically equivalent active sites. This confirms a homogeneous distribution of the adsorbent’s active sites (specifically carboxyl and hydroxyl groups) within the PVC-mesoporous framework, where no further interaction occurs between the adsorbed dye molecules and neighboring sites once the surface reaches saturation. In stark contrast, the adsorption of MB onto the blank PVC film did not fit any isotherm model effectively. The regression coefficients for the blank PVC were remarkably low (R^2^ < 0.66 for Langmuir and 0.2690 for Freundlich), confirming that the unmodified polymer lacks the necessary functional groups and porous architecture for dye sequestration. Notably, the recorded negative value for the Freundlich exponent (1/n = −2.28) and Temkin heat of adsorption (b_T_ = −621.71\ J/mol) for the blank film lack physical significance in classical adsorption theory. These statistically insignificant parameters signal a lack of affinity and poor regression, highlighting that unmodified PVC is not a viable candidate for dye removal. The significantly higher performance of PVC-B aligns with its superior swelling (488%) and porosity (73%), as well as the higher density of pectic functional groups provided by the banana waste component. Furthermore, the adsorption intensity (n) values derived from the Freundlich model were significantly greater than unity (7.0 for PVC-B and 5.03 for PVC-P), indicating highly favorable adsorption conditions. The validity of the D-R model suggests that the adsorption process also involves the filling of the pore volume. This indicates that the diffusion of dye molecules into the porous structure of the composite film is a favored mechanism. This is further supported by the Temkin isotherm, where the heat of adsorption (b_T_) values (285.88 and 234.11 J/mol) suggest a strong electrostatic interaction between the MB cations and the anionic surface. Additionally, the mean free energy (E) derived from the D-R model (668.5 J/mol for PVC-B and 246.2 J/mol for PVC-P) remains well below the 8 kJ/mol threshold. This substantiates that while the process involves specific site binding, it is governed by physical forces such as electrostatic attraction and hydrogen bonding. In conclusion, the synergy between the polymer matrix and the agro-waste additives creates a structured surface that favors Langmuir-type monolayer sequestration combined with micropore filling, a phenomenon consistent with previous reports on modified bio-polymeric^67,68^.

### Adsorption kinetic

Experimental kinetic data for Methylene Blue (MB) adsorption onto the PVC, PVC-B, and PVC-P films were analyzed using pseudo-first-order (PFO), pseudo-second-order (PSO), Elovich, and intraparticle diffusion (IPD) models. Linear and non-linear regressions were applied, with model adequacy assessed via the coefficient of determination (R^2^) and parameter physical realism (Fig S7 and S8) and Table 3. The results indicated chemisorption as the prevailing mechanism across all films, modulated by surface heterogeneity and additive effects.

• Blank PVC film: The linear Elovich model yielded the superior fit (R^2^ = 0.959; α = 0.213 mg/g·min, β = 0.181 g/mg), indicative of chemisorption on a heterogeneous surface with varying activation energies. The linear PSO model performed poorly (R^2^ = 0.133), producing an unphysical negative qe due to linearization errors, consistent with observations on rigid polymers (Sparks, 2003).

• PVC-B film: Non-linear PFO (R^2^ = 0.942; qe = 25.03 mg/g, k₁ = 0.0099 min⁻^1^) and PSO (R^2^ = 0.942; qe = 35.32 mg/g, k₂ = 0.0002 g/mg·min) provided an equivalent excellence, corroborated by the linear Elovich (R^2^ = 0.945). This suggested a synergistic physisorption-chemisorption process, facilitated by banana-derived functional groups and porosity.

• PVC-P film: Linear PSO offered the best fit (R^2^ = 0.992; q_e = 15.34 mg/g, k₂ = 0.0021 g/mg·min), confirming chemisorption-likely via electrostatic interactions and complexation with polyphenolic moieties as the rate-determining step. Supporting evidence from non-linear Elovich (R^2^ = 0.928) underscores the surface heterogeneity^69^. These fits collectively affirm chemisorption via the valence electron exchange over the physisorption or diffusion alone.

The Elovich model parameters further distinguish the additives. The PVC-P film showed a higher initial adsorption rate (α= 1.66 mg/g·min) compared to the PVC-B film (α= 0.313 mg/g·min). This, combined with the high R^2^values, confirmed that the chemisorption on heterogeneous surfaces is a relevant mechanism across all films, though most pronounced in the Pomegranate-doped. IPD analysis revealed multi-linear regimes with non-zero intercepts (C = −4.49 to 5.44 mg/g), implicating boundary layer diffusion alongside intraparticle transport. PVC-Banana displayed the highest k_id (1.661 mg/g·min⁰·^5^), while PVC-Pomegranate’s elevated C (5.445 mg/g) indicated pronounced film resistance^70^.

## Thermodynamic of adsorption

**Fig. 6:**
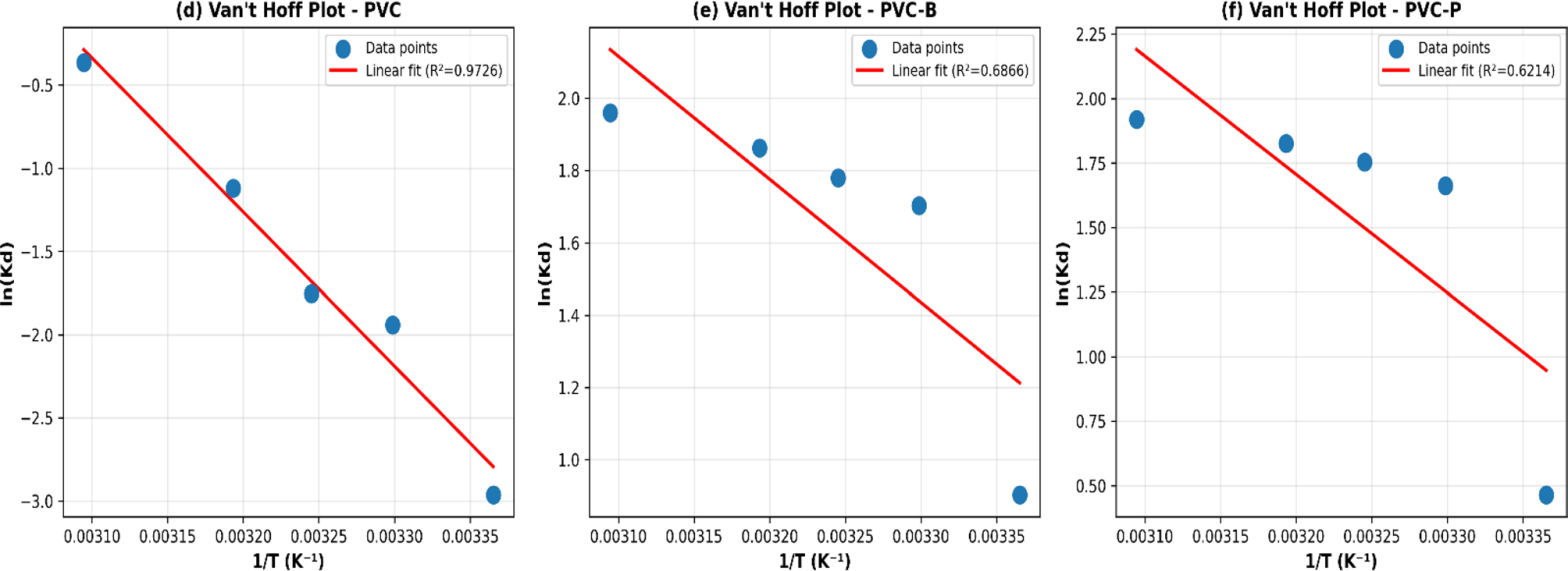
Adsorption thermodynamic of the MB using the PVC, PVC-B, and PVC-P films.

The equilibrium data were analyzed at 298.15 K, 308.15 K, and 318.15 K. The thermodynamic parameters, including standard enthalpy change (ΔHº), standard entropy change (ΔSº), and standard Gibbs free energy change (ΔGº), were derived from the Van 't Hoff equation and are summarized in (Table 4 and Fig. 6).The strong reliability of the obtained results is evidenced by the high coefficients of determination (R^2^ ranging from 0.9958 to 0.9982) from the Van 't Hoff plots, reinforcing the validity of the linear thermodynamic models for this adsorption system. In contrast, models like the Temkin isotherm yielded exceptionally poor fits for the blank PVC (R^2^ = 0.1332), making any derived constants (b_T_) statistically invalid and physically insignificant. Therefore, the thermodynamic analysis provides the definitive description of the process energetics. As shown in Table 4, the values of ΔGº were negative for the blank PVC, PVC-B, and PVC-P composite films, indicating that the adsorption of MB is a spontaneous and highly favorable process. At 298.15 K, the ΔGº values for the composite films (PVC-B: −7.05 kJ/mol; PVC-P: −8.02 kJ/mol) showed superior spontaneity compared to the blank PVC (−6.23 kJ/mol), highlighting the effectiveness of the agro-waste additives. Notably, the ΔGº values became less negative as the temperature increased for all biosorbents. This behavior is characteristic of exothermic reactions, where the adsorption process becomes less spontaneous at higher temperatures; consequently, lower temperatures are likely to enhance the adsorption efficiency for MB removal in these systems. The standard enthalpy change (ΔHº) was negative for all films, ranging from −9.63 to −17.20 kJ/mol, which designates that the adsorption reaction is exothermic in nature. The magnitude of ∆H^o^ (all values < 20 kJ/mol) indicates that the process is primarily governed by physical adsorption involving electrostatic attractions and hydrogen bonding between the MB cations and the anionic functional groups of the biosorbents. Additionally, the negative values of standard entropy (∆S^o^) (−11.40 to −30.79 J/mol·K) indicate a decreased randomness and disorder at the solid-liquid interface during the adsorption of aqueous dye molecules. This trend suggests that the MB molecules adopt a more ordered and structured arrangement upon binding to the surface of the agricultural waste-incorporated films. This decrease in disorder was most pronounced for the PVC-P film (−30.79 J/mol·K), which also exhibited the highest spontaneity, confirming that the incorporation of pomegranate waste provides the most favorable energetic conditions for effective dye sequestration.

## Adsorption mechanism

**Fig. 7:**
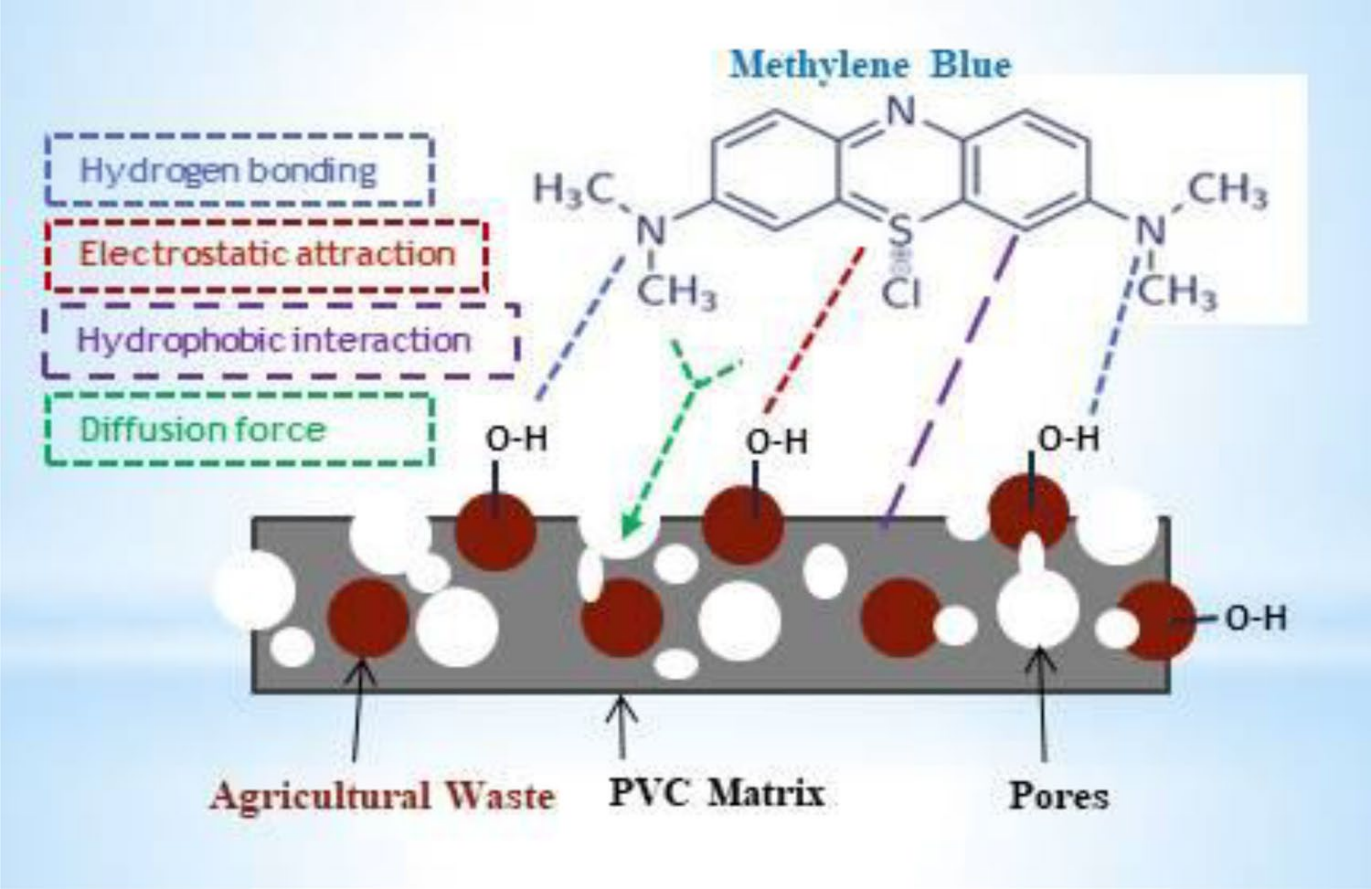
proposed mechanism for the adsorption of MB using the PVC, PVC-B, and PVC-P films.

The proposed mechanism for the adsorption of methylene blue dye onto PVC-B and PVC-P composite films include different approaches such as hydrogen bonding among reactive functional groups such as hydroxyl present in the polysaccharide structure of agricultural wastes and the dye molecules, also the electrostatic interaction between the charged molecules of MB dye and polar groups on the biomass structure^71^. Also the adsorption may include a hydrophobic interaction between the benzene and aromatic rings of the MB dye molecules and hydrophobic chains of polyvinyl chloride, and intra-diffusion and interpenetration of the MB molecule into the internal pores within the polymer matrix (Fig. 7) in addition of weak Vander Waals attraction forces^19^.

## Desorption studies

**Fig. 8:**
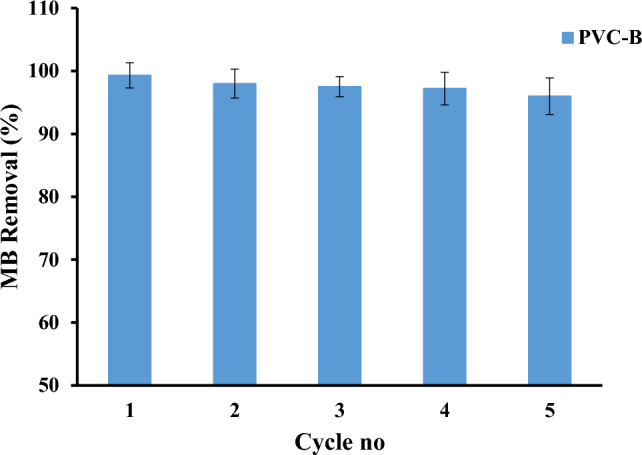
Desorption studies of the MB by using the PVC-B film.

Desorption experiments were successfully applied using the best run obtained from the full factorial design experiment where the PVC-B film was used for 50 ppm MB concentration removal where the contact time was 180 min. Five adsorption - desorption cycles were achieved using 50 % aqueous ethanol solution as a washing media. It is well known that ethanol is highly efficient to desorb dye from an adsorbent material. Also aqueous ethanol was chosen as it is an ecofriendly solvent, non-corrosive and can be recycled by simple distillation The existence of ethanol in water produced a decline in surface tension, which helpful in the movement of pollutant molecules from the adsorbent matrix to the bulk of solution in dye desorption step and can enhance the ionization and solubilization of the dyes^72^. It was noticed (see Fig. 8**)** that, the efficiency of composite film in MB removal was 96 % of its initial value after five cycles of adsorption desorption process. This result may be attributed to the effect of ethanol that, it worked as a pre-treatment for the films and can facilitate a new site for MB removal by breaking the inter-hydrogen bonding between the cellulosic molecules within the agricultural waste and made them available for adsorption. The results of regeneration process indicate that the composite film is a good candidate in dye removal application for several time without significance loss of it efficiency.

## Cost evaluation analysis

A preliminary quantitative cost evaluation was carried out to support the identification of the proposed PVC-B and PVC-P composite films as inexpensive environmental biosorbents. In order to maintain a 10 wt/v% polymer concentration, 10 g of commercial PVC powder must be dissolved in 90 mL of Dimethylformamide (DMF) solvent (~85.5 g based on DMF density of 0.944 g/mL). Next, 10 wt% of finely ground agro-waste particles (1 g of sieved banana or pomegranate peel) must be blended in relation to the polymer weight. The agricultural wastes were purchased locally from marketplaces in Cairo, Egypt, at no cost for raw materials, and only a small amount of energy was needed for ball milling and oven drying (90℃ for 24 hours). The estimated prices are based on current industrial/bulk chemical market values per kilogram

As shown in Table S1, the solvent (DMF) is the main expense in the baseline cost of $19.51 to synthesis 1 kg of dry pristine PVC film using the phase inversion casting technique. The direct raw material fabrication cost decreases to $17.75 per kilogram when 10 weight percent of agricultural waste is added to the polymer (equivalent to an overall composition of 90.9% PVC and 9.1% agro-waste in the final dry film grid), which represents a 9.02% reduction in total chemical expenses. Standard fractional distillation can economically recover up to 80–85% of the DMF solvent used in the non-solvent coagulation bath (water) in an industrial setting. The real manufacturing cost of the composite films significantly decreases to about $4.69/kg for PVC-B/PVC-P under an 80% solvent recovery system, as opposed to $5.14/kg for the blank PVC.

By comparing these expenses with the maximum adsorption capacities (q^max^) derived from the Langmuir fitting, the ultimate economic viability is demonstrated. The blank PVC film has an insignificant capacity of 7.73 mg/g, but adding merely 10 weight percent agro-waste increases the capacity to 79.89 mg/g (for PVC-B) and 70.92 mg/g (for PVC-P), resulting in an exceptional 933% and 817% performance rise, respectively. As a result, the composite films have an order of magnitude reduced cost per unit of dye removed.

In contrast to commercial high-grade granular activated carbon (GAC), which has a market price range of $2.50 to $4.50/kg but frequently lacks the membrane-like reusability and flexible mechanical integrity of casted polymer films, the developed bio-composites represent a highly competitive, environmentally friendly alternative that is in line with the circular economy and green innovation principles.

## Conclusion

Three types of polymer films; Polyvinyl chloride (PVC) as a blank polymer, PVC-Banana (PVC-B), and PVC-Pomegranate (PVC-P) were successfully prepared in order to be used in the MB removal process. The composite films were prepared using phase inversion technique and the morphological study showed the highly porous surface with macrovoides cross-section structure. Chemical and physical investigation indicated the high swelling ratio reached 488% with porosity of about 70%. The composite films were employed in MB dye removal from waste water. The OFAT trials and full factorial design were performed successfully for obtaining the optimum conditions that achieve high MB removal efficiency. Results showed that the MB removal efficiency of 94 and 83% was achieved by using 4 g/l from PVC-B and PVC-P composite films, respectively, at pH 7 and 100 ppm MB concentration after 180 minutes of contact time. Adsorption of the MB using PVC-B, and PVC-P films fitted well with Langmuir and D-R isotherm models indicating the chemical nature of adsorption accompanied by filling mechanism in the micro-porous voids within the polymer matrix. Kinetics studies indicated the adsorption was more fitted to pseudo second order and Elovich kinetic models confirming the chemo-sorption on heterogeneous surfaces mechanism. Regeneration studies suggested that, the PVC-B films can be used for five respective cycles where the film preserved its efficiency for all cycles where it achieved around 95 % in the 5^th^ run. From the results, it can be stated that the applicability and efficiency of using PVC-B film for the MB removal from wastewater is a cheap and eco-friendly approach. For further investigation in the future, the films can be used through a reactor design in order to ease the application of the removal process in the industrial sector.

## Supplementary Information

Supplementary Information.

## Data Availability

The datasets used and/or analyzed during the current study available from the corresponding author on reasonable request.
