# Supplementary material for: Sustainable composites based on banana and pomegranate waste incorporated into Polyvinyl Chloride Matrix for methylene blue adsorption
Source: Sci Rep. 2026 Jun 8;16:17698. doi: 10.1038/s41598-026-55367-2 (PMC13247246; doi:10.1038/s41598-026-55367-2)
Supplement: Supplementary file 1 — Supplementary Information. [file 41598_2026_55367_MOESM1_ESM.docx]

Fig S1


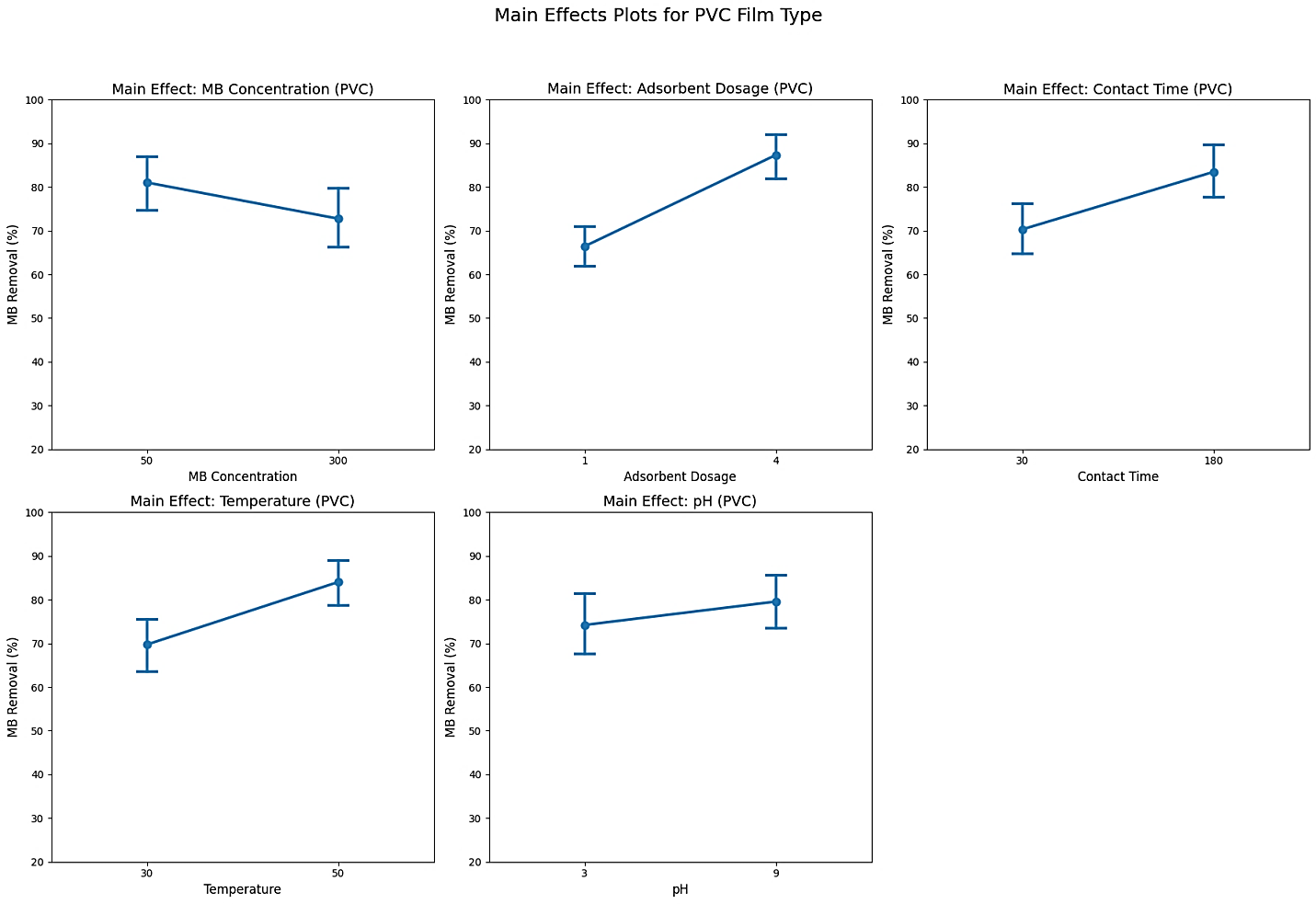


**Fig S2-a**


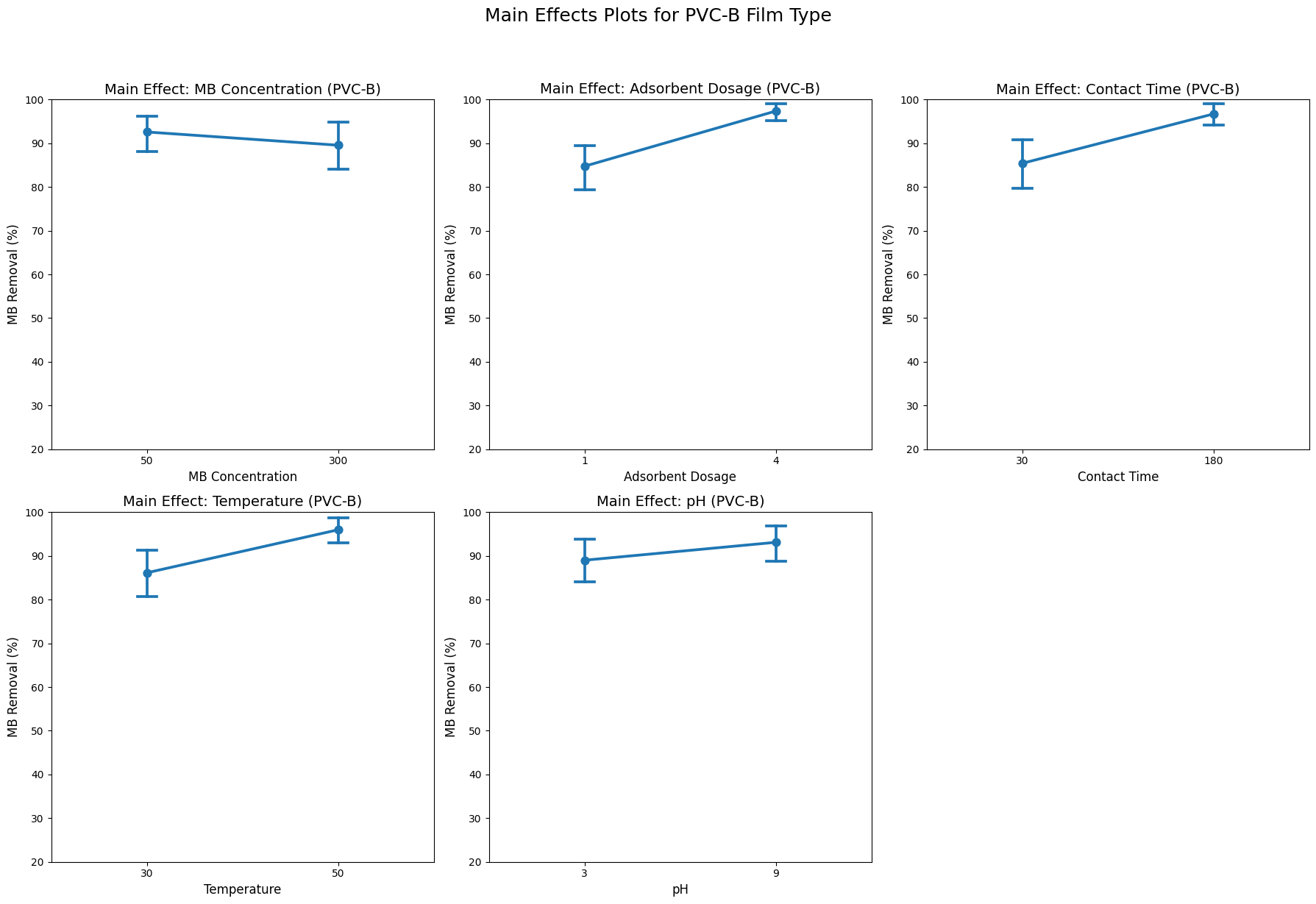


**Fig S2-b**


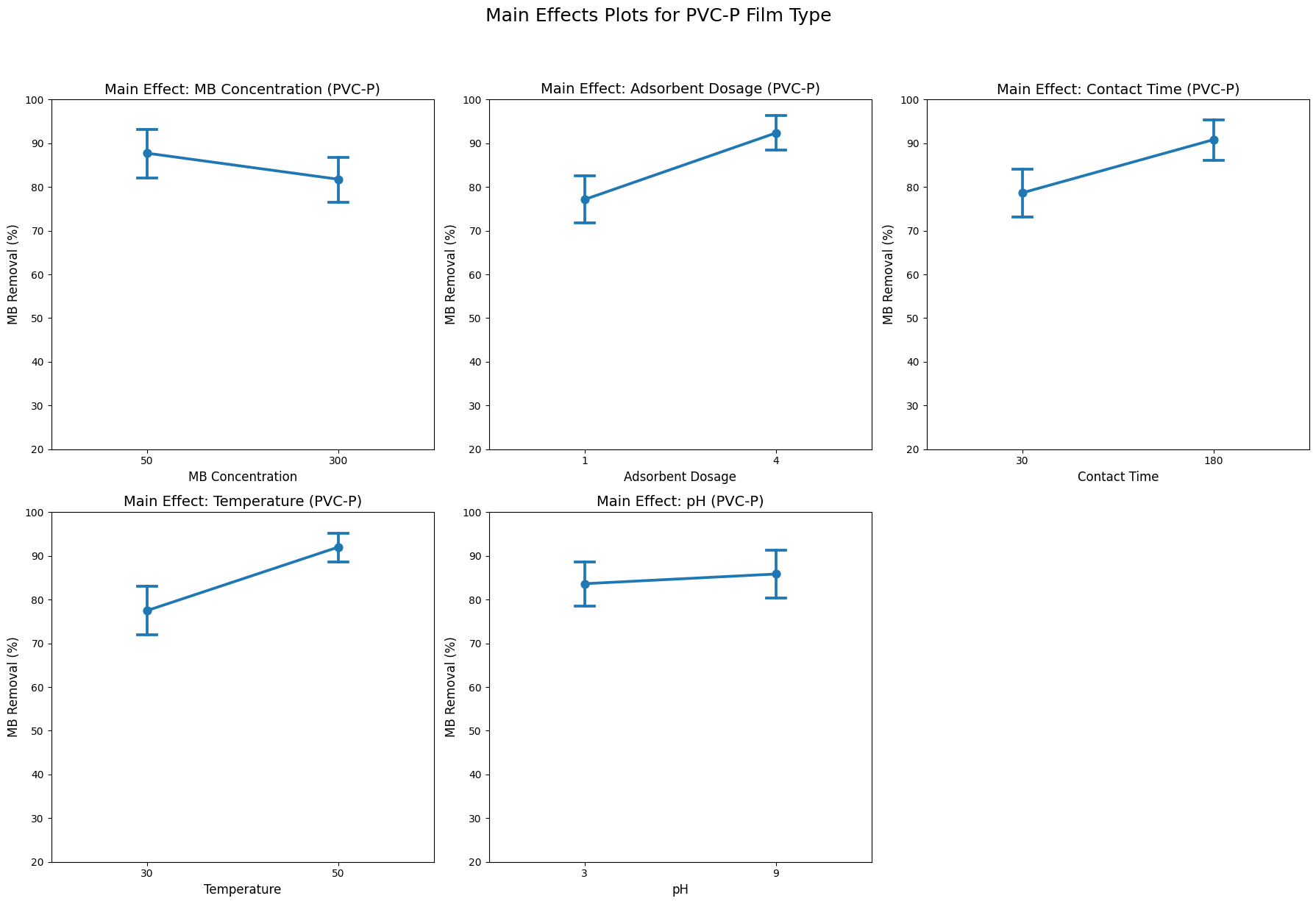


**Fig S2-c**

**Fig S2 (a, b & c)**


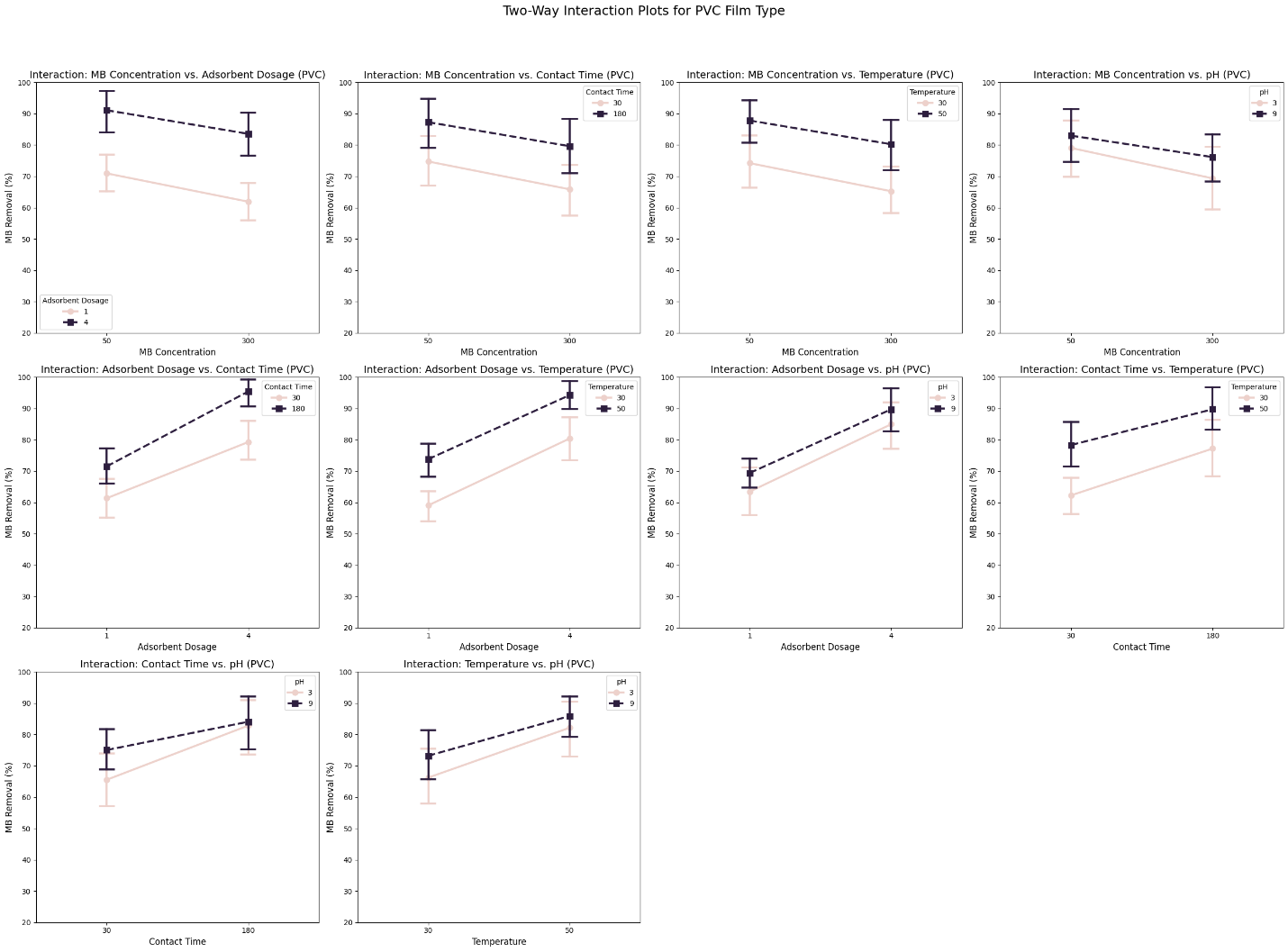


**Fig S3-a**


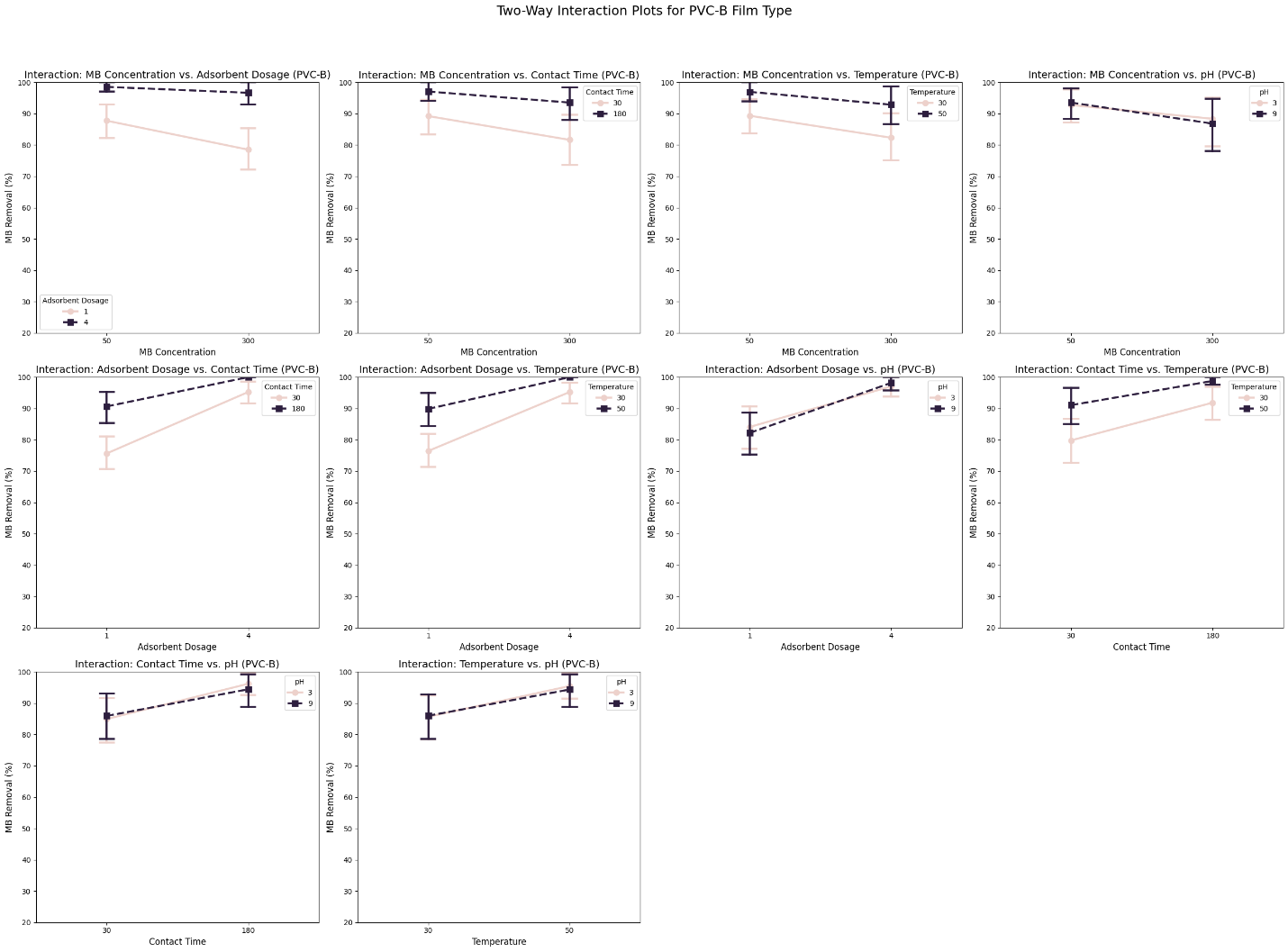


**Fig S3-b**


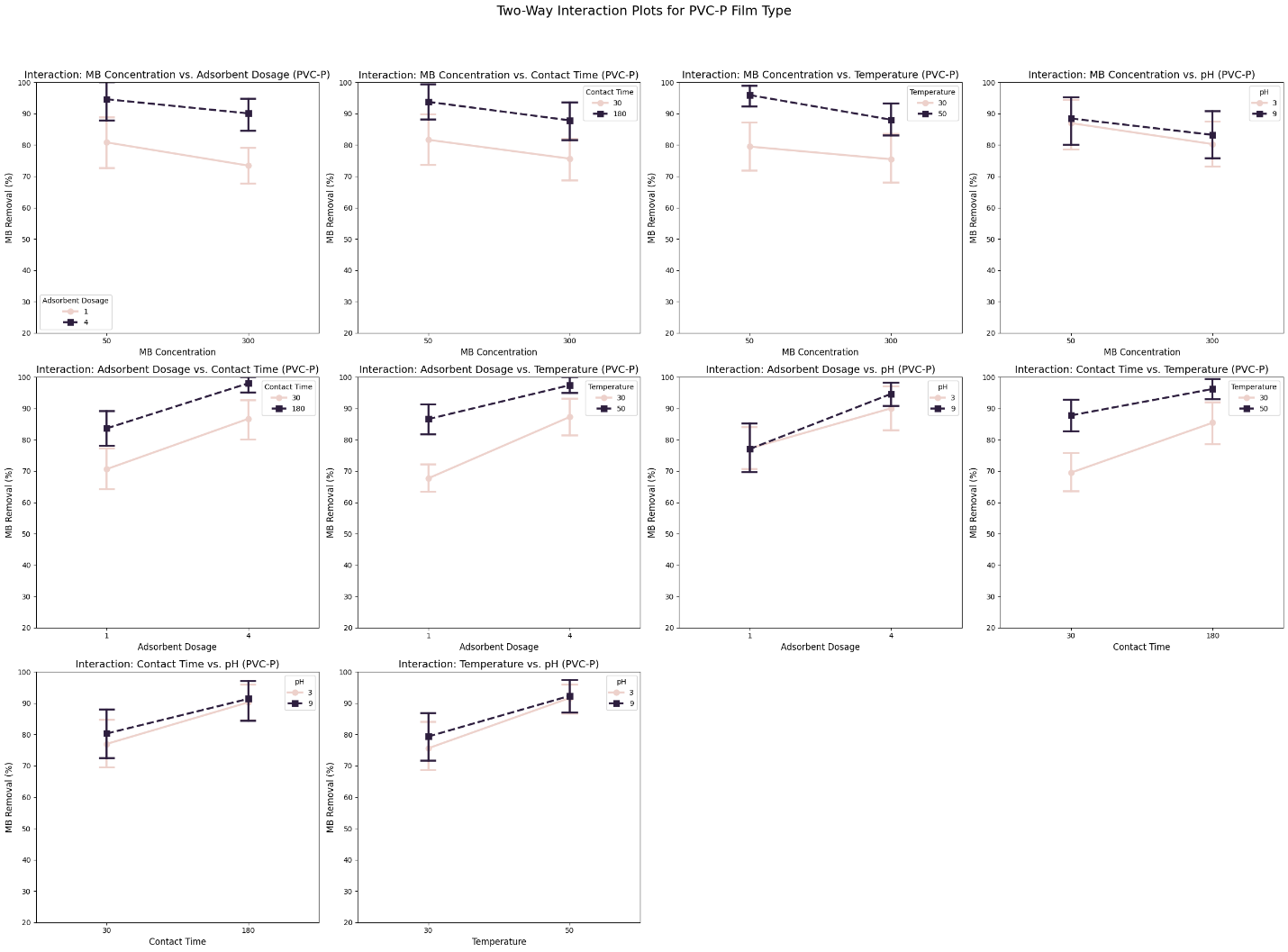


**Fig S3-c**

**Fig S3 (a, b & c)**


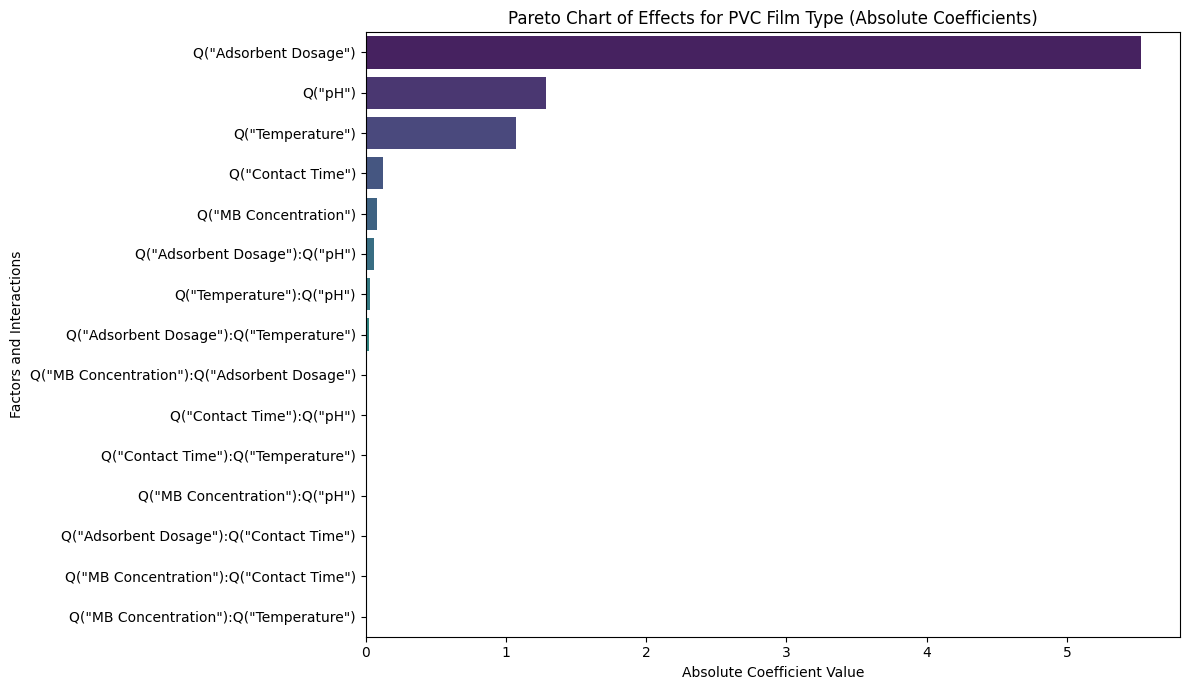

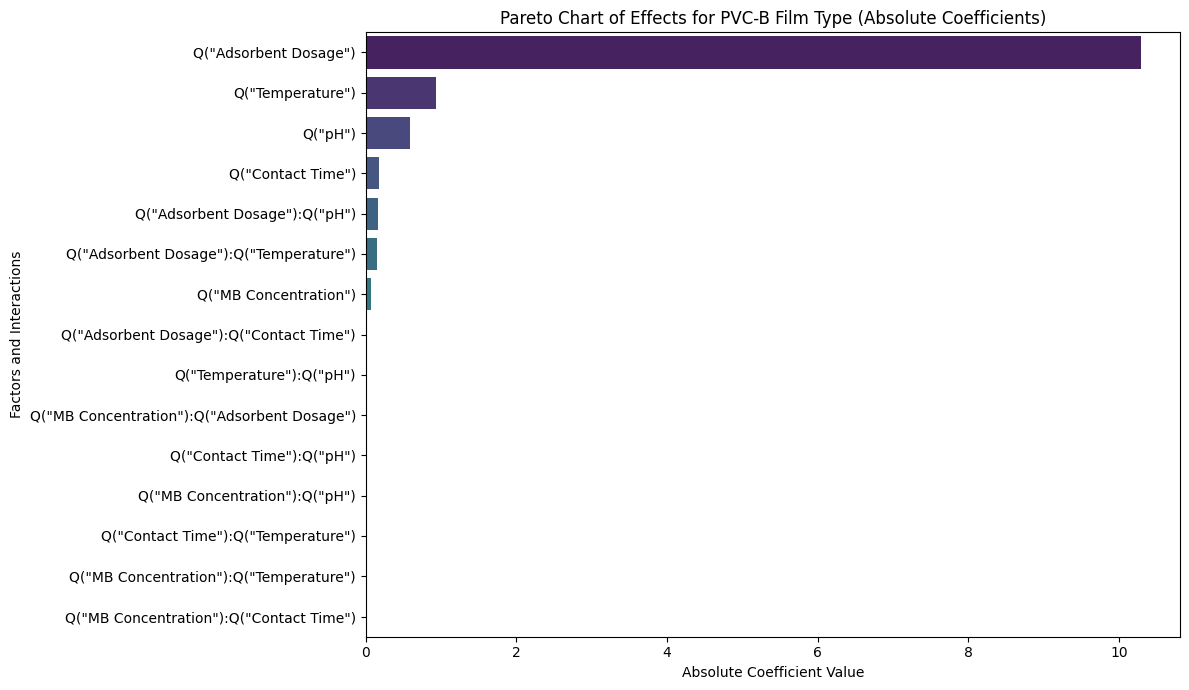

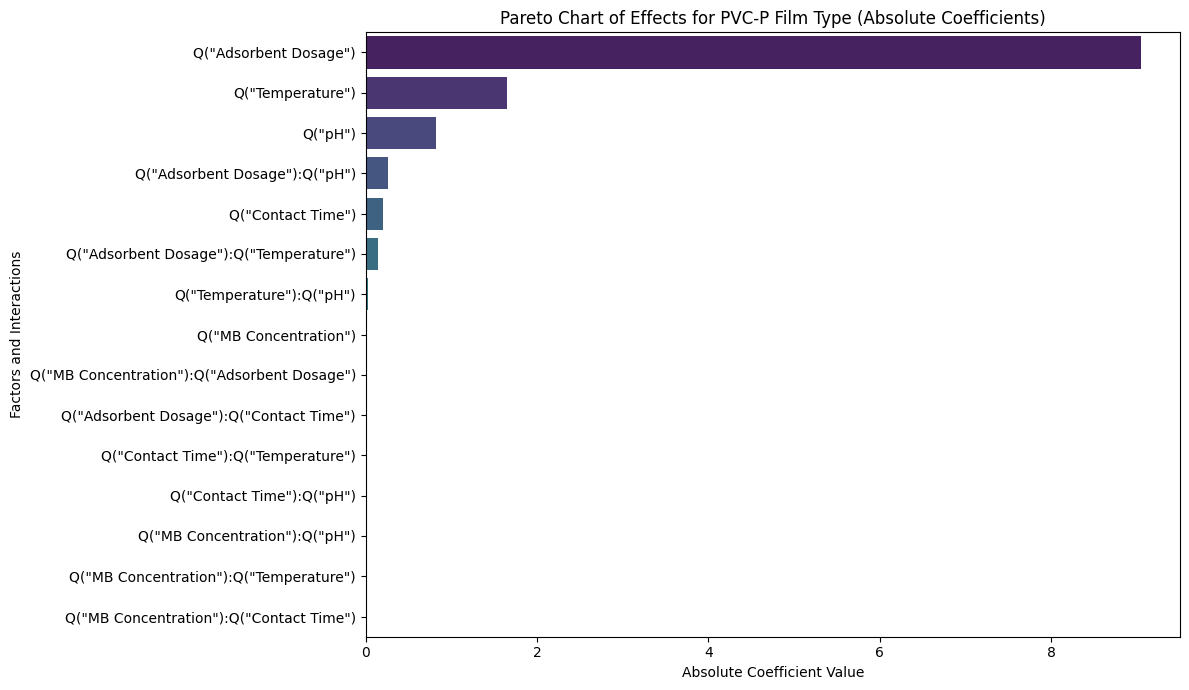


**Fig S4-b**

**Fig S4-a**

**Fig S4-c**

**Fig S4 (a, b & c)**

**a**

**b**

**Fig. S5 (a & b)**

**c**

**d**

**Fig. S5 (c & d)**

**a**

**b**

**Fig. S6 (a & b)**

**c**

**d**

**Fig. S6 (c & d)**


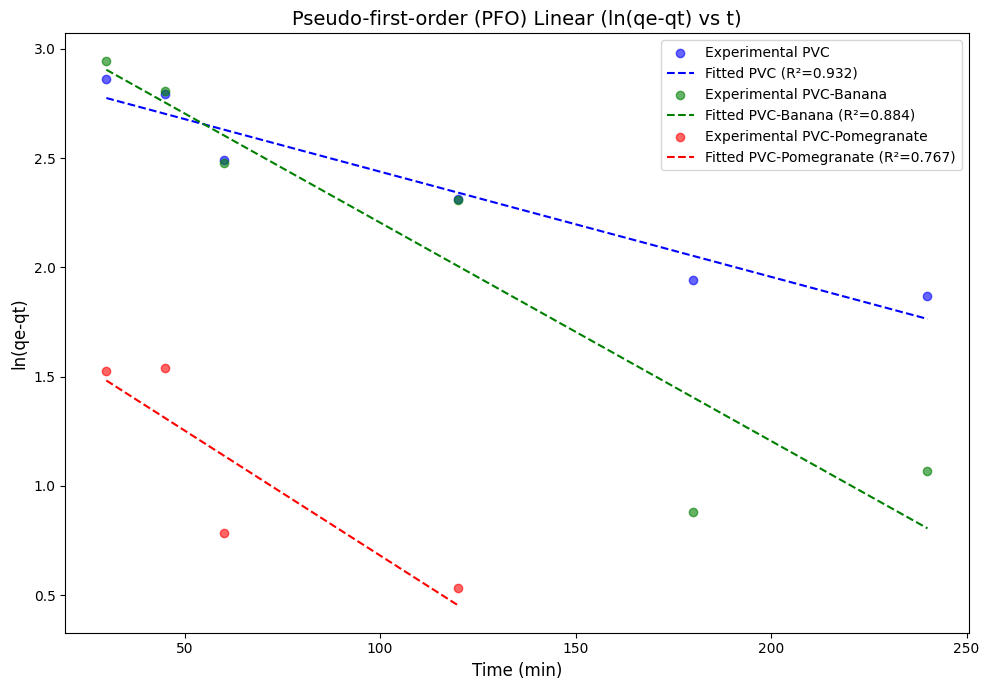


**a**

**b**


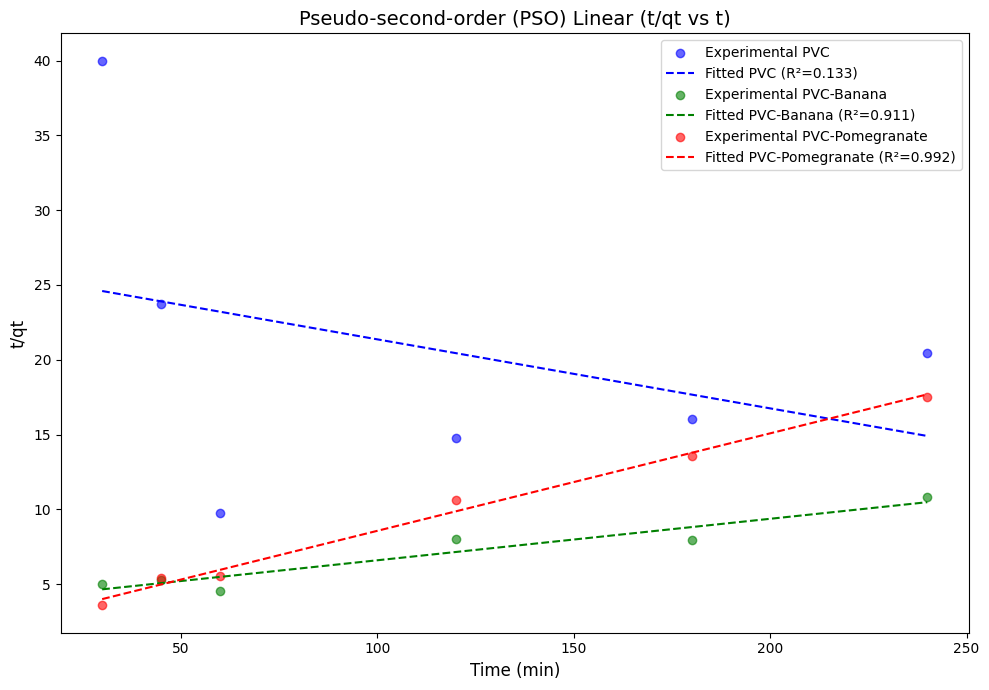


**Fig. S7 (a & b)**

**
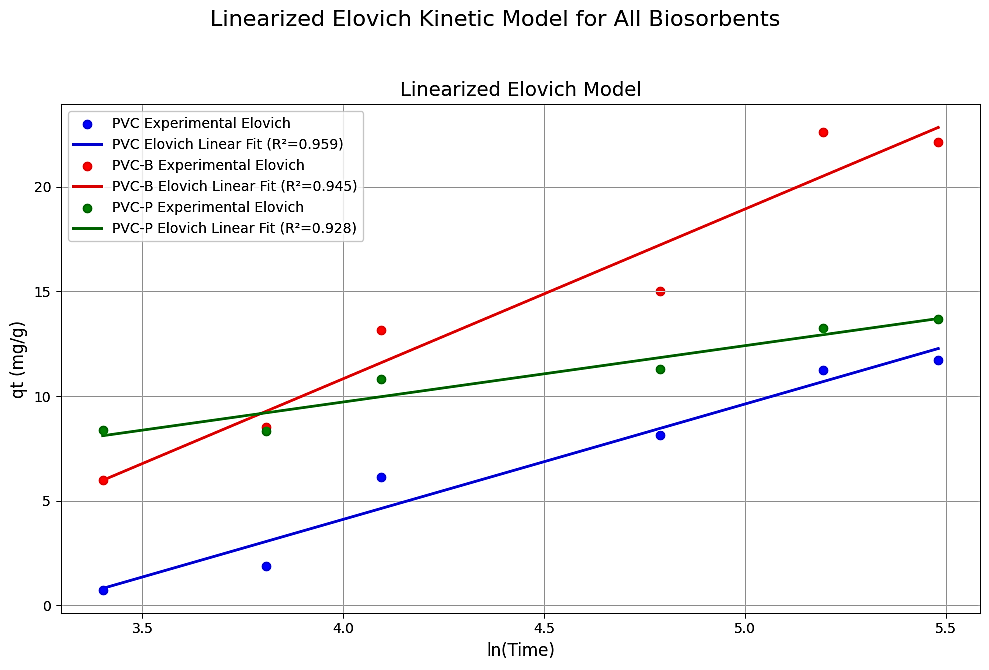
**

**c**

**
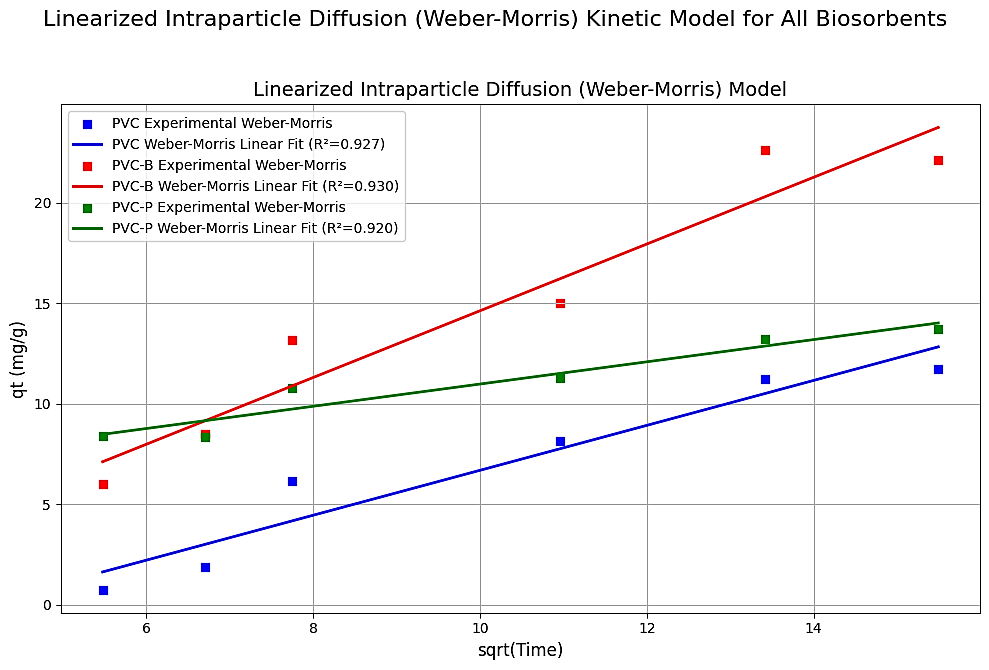
**

**d**

**Fig. S7 (c & d)**


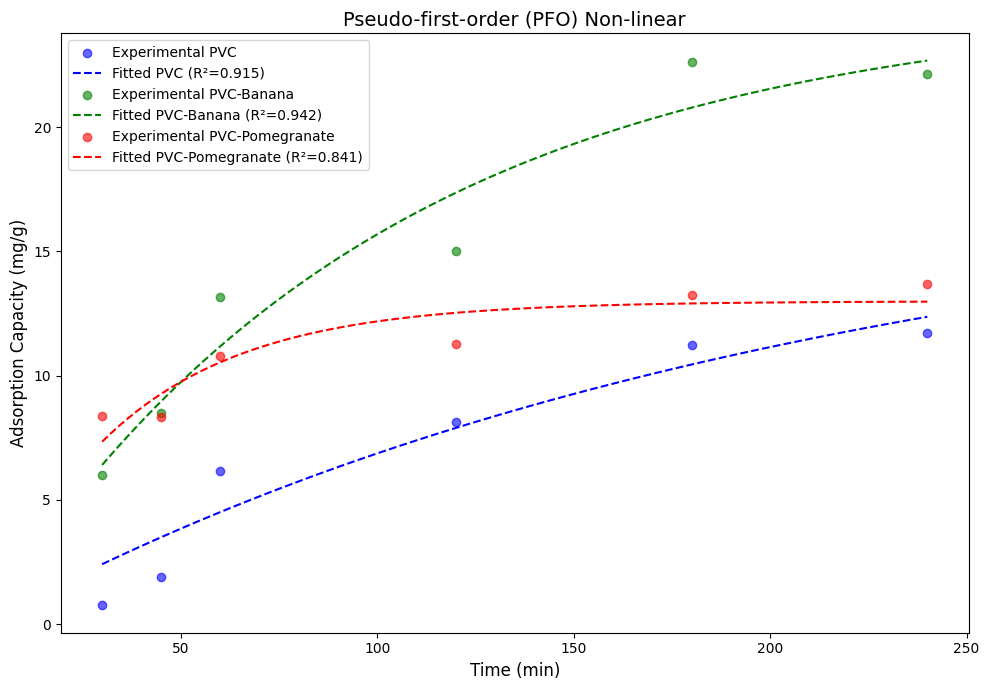


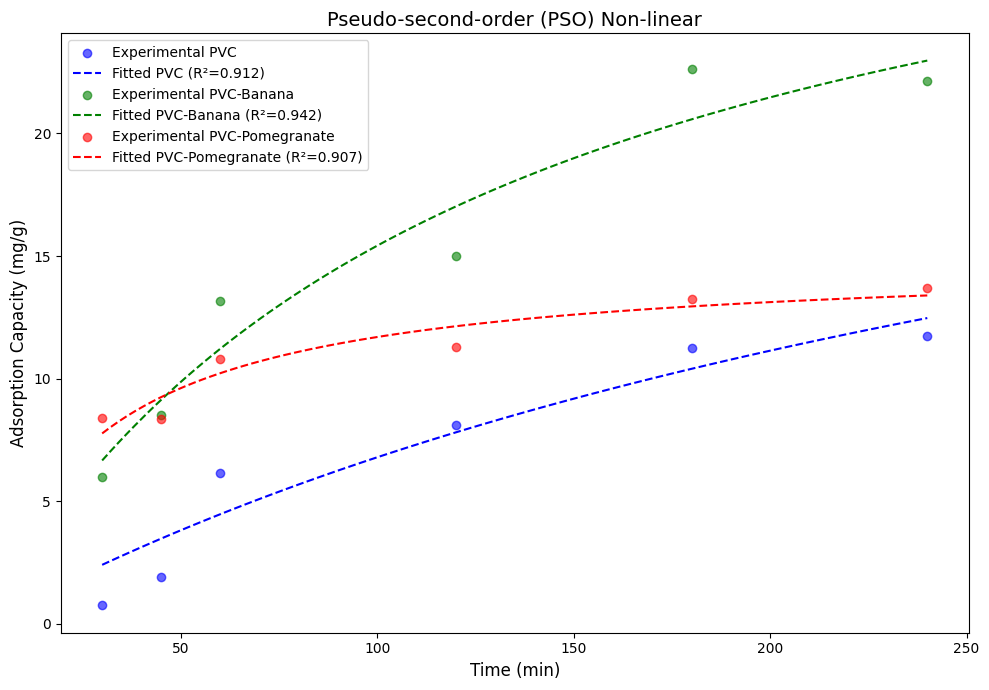


**Fig. S8 (a & b)**


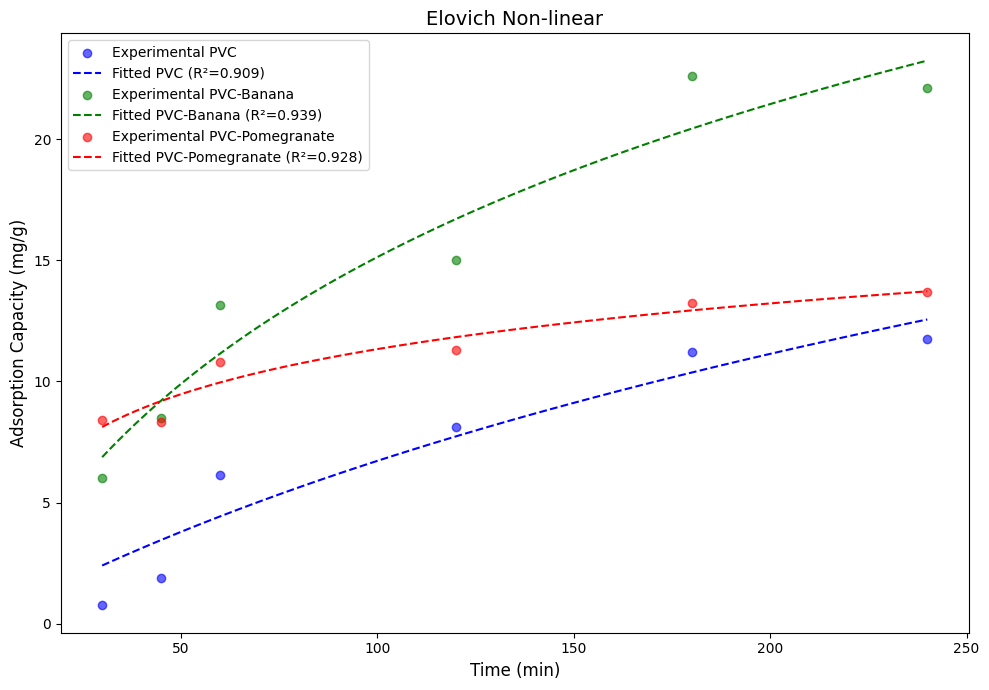


**
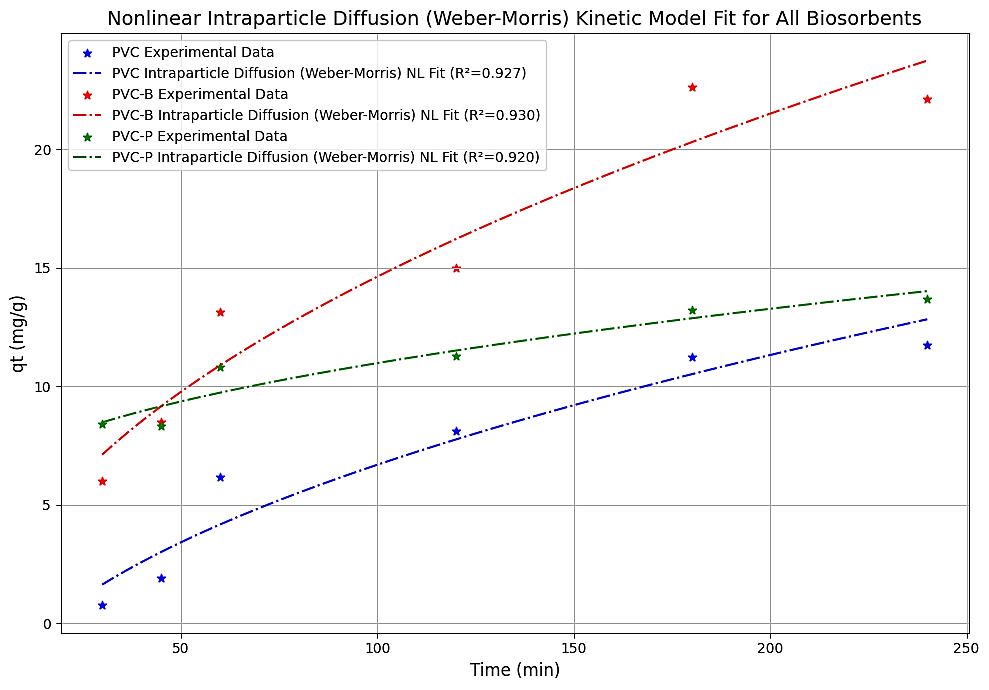
**

**Fig. S8 (c, & d)**

**Table S1. Preliminary raw material cost estimation for fabricating 1 kg of the adsorbent films.**

| **Component Process** | **Bulk Unit Cost (USD/kg)** | **Mass for Blank PVC Film (kg)** | **Mass for PVC-B / PVC-P Film (kg)** | **Cost for Blank PVC Film (USD)** | **Cost for PVC-B / PVC-P Film (USD)** |
| --- | --- | --- | --- | --- | --- |
| **Commercial PVC Powder** | **$1.50** | **1.000** | **0.909** | **$1.50** | **$1.36** |
| **Dimethylformamide (DMF)** | **$2.10** | **8.550** | **7.773** | **$17.96** | **$16.32** |
| **Agro-waste Peels (Sieved)** | **$0.00** | **0.000** | **0.091** | **$0.00** | **$0.00** |
| **Drying & Milling Energy** | **$0.25** | **0.000** | **0.091** | **$0.00** | **$0.02** |
| **Water (Coagulation Bath)** | **$0.01** | **Variable** | **Variable** | **$0.05** | **$0.05** |
| **Total Fabric Cost/kg** | **—** | **—** | **—** | **$19.51** | **$17.75** |
